# Mushrooms in climate change mitigation: a comprehensive review

**DOI:** 10.3389/fmicb.2025.1727022

**Published:** 2026-02-04

**Authors:** Samantha C. Karunarathna, Saowaluck Tibpromma, Baggya Sharmali Karunarathna, Dong-Qin Dai, Jaturong Kumla, Wenhua Lu, Rekhani Hansika Perera, Meimei Wang, Tikka Dewage Chamarika Priyadarshani, Kalani Kanchana Hapuarachchi, Nakarin Suwannarach

**Affiliations:** 1 Center for Yunnan Plateau Biological Resources Protection and Utilization & Yunnan International Joint Laboratory of Fungal Sustainable Utilization in South and Southeast Asia, College of Biology and Food Engineering, Qujing Normal University, Qujing, China; 2 Department of Chemistry, Faculty of Science, Eastern University Sri Lanka, Chenkalady, Sri Lanka; 3 Center of Excellence in Microbial Diversity and Sustainable Utilization, Chiang Mai University, Chiang Mai, Thailand; 4 Office of Research Administration, Chiang Mai University, Chiang Mai, Thailand; 5 Zest Lanka International (Private) Limited, Wadichchalaya, Polonnaruwa, Sri Lanka; 6 Department of Plant Sciences, Faculty of Agriculture, Rajarata University of Sri Lanka, Anuradhapura, Sri Lanka; 7 College of Biodiversity Conservation, Southwest Forestry University, Kunming, China

**Keywords:** carbon sequestration, ectomycorrhizal symbiosis, extreme environments, heavy metal remediation, lignocellulose degradation, mycoremediation, pollutant degradation

## Abstract

Mushroom-forming basidiomycetes are increasingly recognized for their significant potential to remediate polluted environments and mitigate climate change. This review synthesizes evidence positioning mushroom-forming basidiomycetes at the nexus of ecological resilience and a sustainable bioeconomy, highlighting their dual roles in environmental repair and green innovation. Ectomycorrhizal (ECM species) enhance carbon acquisition by plants and long-term soil carbon sequestration; ECM-dominant forests stockpile upto 70% more below-ground carbon than their non-mycorrhizal counterparts. Saprotrophic fungi drive lignocellulose degradation, nutrient cycling, and the stabilization of soil organic matter. Basidiomycetes also play a crucial role in mycoremediation by degrading recalcitrant contaminants (pesticides, hydrocarbons) and immobilizing heavy metals. Furthermore, mycelium-based biomaterials are being developed as green-technology alternatives to plastics and synthetic foams, reflecting the growing commercialization of fungal biotechnology, as evidenced by the global mycelium material industry projected to exceed USD 5 billion by 2032. The intersection of ecological function and economic value positions mushrooms at the forefront of the circular bioeconomy. However, challenges remain, including production scalability, environmental sensitivity, and economic viability. Addressing these challenges through interdisciplinary research could unlock the full potential of fungi as nature-based climate solutions.

## 1 Introduction

Global warming is the defining challenge of the 21st century, with Earth's surface temperatures repeatedly reaching record highs due to greenhouse gas (GHG) emissions from human activities. Atmospheric CO_2_ concentrations have risen from approximately 280 ppm in pre-industrial times to over 420 ppm as of 2024, primarily due to fossil fuel combustion, agriculture, industry, and deforestation (Nunes, 2023; Hansen et al., 2025). These climatic changes have led to increased weather unpredictability, biodiversity loss, reduced ecosystem resilience, and compromised water and food security. As nature-based solutions gain traction, fungi, particularly understudied mushroom-producing Basidiomycetes, are emerging as a significant resource due to their dual roles in carbon cycling and biotechnological innovation (Muluneh, 2021; Dunlop et al., 2024).

Among diverse life forms, mushroom-forming Basidiomycota are of particular research interest due to their significant potential in mitigating global warming. These fungi are key agents of carbon cycling, environmental detoxification, and sustainable biomaterial production (Fukasawa and Kimura, 2025). In contrast to unicellular yeasts and molds, mushroom-producing basidiomycetes form complex multicellular fruiting bodies and extensive mycelial networks (Martin and van der Heijden, 2024; Sreerag et al., 2025). Despite their crucial ecological roles, mushrooms remain underrepresented in major climate action and restoration strategies, such as the Paris Agreement (2015) and the UN Decade of Ecosystem Restoration (2021–2030), which focus on reforestation, soil preservation, and biodiversity—areas where mycelial networks are fundamentally important [Lofgren and Stajich, 2021; United Nations Economic and Social Commission for Asia and the Pacific (ESCAP), and World Health Organization (WHO), 2022; Niskanen et al., 2023].

Carbon sequestration is a critical ecological function of mushrooms. Mycorrhizal fungi, particularly ectomycorrhizal (ECM) species like *Boletus, Amanita*, and *Laccaria*, form symbiotic associations with tree roots that enhance nutrient exchange and carbon sequestration in forest soils (Nuralykyzy et al., 2025). These associations improve tree growth, soil aggregate stability, and the storage of recalcitrant organic matter (Hawkins et al., 2023; Zhang E. et al., 2025; Zhang X. et al., 2025). Meta-analyses and ecosystem comparisons suggest that forests dominated by ECM symbionts can accumulate substantially more soil carbon—in some reports up to 70% more, than forests dominated by arbuscular mycorrhizal (AM) trees (Averill et al., 2014). However, this difference represents a broad ecological pattern that emerges at the biome scale and is influenced by multiple co-varying factors, including litter quality, nitrogen availability, and climate. The specific, mechanistic contribution of ECM fungi to this carbon stock—distinct from the traits of their host trees—remains an active area of research and is difficult to isolate in field studies. Modeling and synthesis efforts have led to the influential estimate that fungal processes mediate 15–20% of terrestrial annual carbon sequestration (Wang et al., 2023; Yang et al., 2024; de Goede et al., 2025). This figure, however, represents a global-scale extrapolation from heterogeneous site-level data and model outputs. Its reliability is contingent on parameters that are difficult to constrain universally, such as the turnover rate of fungal necromass and the stability of mineral-associated organic carbon (MAOC) derived from fungal activity across different biomes.

Beyond symbiotic fungi, saprotrophs like *Pleurotus ostreatus, Ganoderma lucidum*, and *Agaricus bisporus* decompose recalcitrant plant polymers such as lignin and cellulose, thereby facilitating organic matter turnover and nutrient cycling (Stajić et al., 2022; Dou et al., 2025). These mushrooms break down the sequestered carbon in plant residue from crops and woody vegetation using oxidative enzymes, including multifunctional peroxidases, manganese peroxidases (MnP), and laccases (Xu et al., 2017; El-Ramady et al., 2022; Nwagu et al., 2025). By breaking down recalcitrant biomass into simpler compounds, saprotrophs enhance soil fertility and secondary productivity (Hu et al., 2021; Chaudhari and Sonawane, 2025). In addition to carbon cycling, mushrooms play a significant role in environmental detoxification. *Trametes versicolor* and *P. ostreatus*, for example, exhibit white-rot fungal properties and possess enzymatic capabilities that enable them to break down xenobiotic molecules, including petroleum hydrocarbons, polychlorinated biphenyls (PCBs), dyes, and pesticides (Akpasi et al., 2023; Costa et al., 2025; Khan, 2025). Their ligninolytic enzymes can degrade high redox potential non-phenolic pollutants, making them highly suitable for mycoremediation (Zhuo and Fan, 2021; Daâssi et al., 2025). Furthermore, mushrooms like *Lentinula edodes* and *Ganoderma applanatum* can immobilize and bioaccumulate toxic heavy metals, such as lead, cadmium, and arsenic, in contaminated soil, thereby facilitating ecosystem detoxification and remediation (Ipeaiyeda et al., 2020; Acharya et al., 2025).

Beyond their well-established ecological functions, mushrooms are now recognized as engines of a new bio-based economy, with their mycelial networks offering platform technologies for environmental and industrial applications. Mycelium, the vegetative and non-reproductive phase of a fungus, has been engineered into biodegradable products used in packaging, construction, and fashion. Mycelium composites are carbon-neutral, fire-resistant, durable, and dense, meeting circular economy standards (Barta et al., 2024; Xia, 2024). Global demand for mycelium-based products is projected to exceed USD 5 billion by 2030, driven by growing demand in green construction, insulation, and fashion (Grand View Research, 2024). Additionally, fungal biomass is a promising feedstock for next-generation biofuels; lignocellulosic agricultural residues depolymerized by fungal enzymes can be fermented into bioethanol, which has a significantly lower life-cycle greenhouse gas emission profile than fossil fuels (Devi et al., 2022; Ilić et al., 2023; Yin et al., 2025).

Nevertheless, despite this promise, the large-scale application of mushroom-based technologies faces several challenges. These include the sensitivity of fungi to environmental growth conditions, variability in enzymatic expression, substrate availability, competition with indigenous microbiota, and the current economic non-viability of large-scale mycelium material production (Le Ferrand, 2024; Wattanavichean et al., 2025). Furthermore, contemporary research is often fragmented across disciplines such as environmental engineering, materials science, and fungal ecology, hindering the development of integrated climate solutions (Case et al., 2022; Srivastava et al., 2025). Many studies have focused narrowly on a single application (e.g., mycoremediation or mycomaterials), overlooking the potential synergies between ecological and biotechnological services that could position mushrooms as major contributors to climate mitigation (Akhtar and Mannan, 2020; Jin et al., 2025).

**Figure 1:**
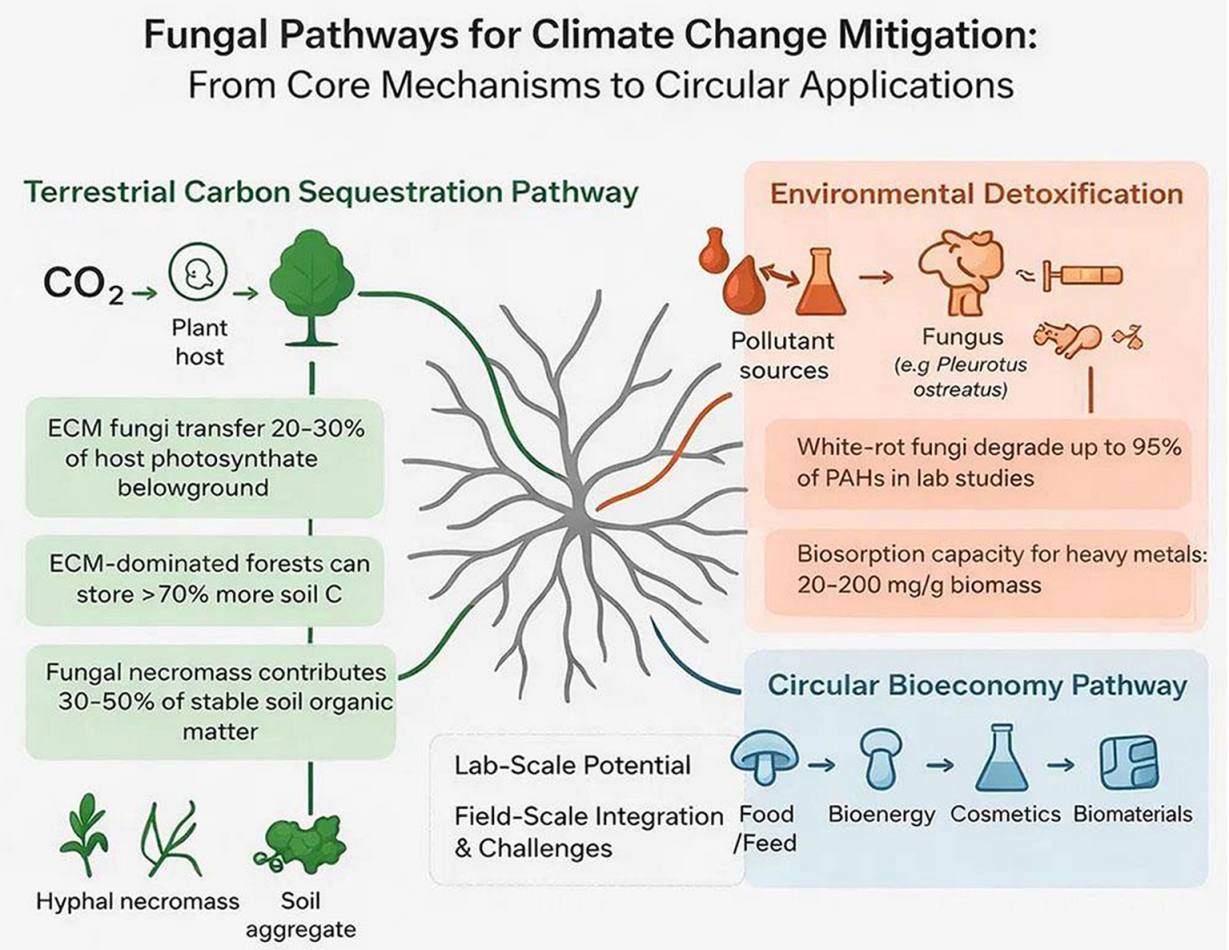
Conceptual overview of fungal pathways for climate change mitigation, integrating core ecological mechanisms with circular bioeconomy applications.

This review focuses on the two most critical mechanisms by which mushrooms mitigate climate change: (1) their role in terrestrial carbon sequestration and stabilization, and (2) their enzymatic degradation of persistent pollutants. We then synthesize how these mechanisms underpin applied technologies in bioenergy and green materials. By focusing on these core pathways, we provide a focused analysis of fungi as essential agents for climate-resilient ecosystems and a low-carbon future. The interlinked ecological and biotechnological pathways through which fungi contribute to climate change mitigation are summarized in Figure 1.

## 2 Core mechanism I: carbon sequestration and soil carbon stabilization

Fungi mitigate climate change through interconnected biochemical mechanisms. This section examines two core processes: the stabilization of atmospheric carbon into long-term soil pools (Section 2.1) and the enzymatic breakdown of organic matter that regulates carbon and nutrient cycles (Section 2.2), highlighting functional roles over taxonomic categories.

### 2.1 Mycorrhizal and saprotrophic pathways for soil carbon storage

Mushrooms are key regulators of carbon cycling in terrestrial ecosystems, influencing both short-term turnover and long-term storage (He et al., 2023; Chen B. D. et al., 2024). The symbiosis between plants and mycorrhizal fungi, particularly species like *Boletus, Amanita*, and *Laccaria*, enhances photosynthesis and biomass accumulation in the host plant by facilitating nutrient transfer. This symbiosis is an important contributor to organic carbon sequestration within forest soils through the production of recalcitrant material and soil aggregates (Vaario and Matsushita, 2021; El Amrani, 2024). In contrast, saprotrophs like *Pleurotus* and *Ganoderma* break down lignocellulosic residues, thereby driving organic matter turnover. Through the degradation of complex plant polymers like lignin and cellulose, they maintain a balance between carbon sequestration and release, ultimately promoting soil fertility and long-term carbon sequestration (Ogwu et al., 2025a; Nuralykyzy et al., 2025). The synergy between these two ecological functions, decomposition and symbiosis, is fundamental to leveraging fungi in nature-based climate change mitigation. Mycorrhizal fungi form symbiotic associations with plant roots that enhance long-term nutrient and carbon storage. Their extensive hyphal networks help form stable organic carbon compounds, reduce CO_2_ emissions, and promote carbon sequestration in forest ecosystems (Averill et al., 2014; Yang et al., 2024). The two main types, arbuscular mycorrhizal (AM) and ectomycorrhizal (ECM) fungi, both contribute significantly to soil fertility and carbon sequestration in forests.

#### 2.1.1 Ectomycorrhizal mushrooms and carbon stabilization in forest soils

Studies of specific tree-fungus pairs, such as those involving *Pisolithus tinctorius* or *Laccaria bicolor*, indicate that ECM hyphal networks can channel 20–30% of the carbon fixed by their host plant belowground (Cope et al., 2019). This range stems primarily from controlled greenhouse experiments using young seedlings in simplified, often semi-sterile systems. In mature forests, this allocation percentage is dynamic and varies significantly with tree age, season, soil nutrients, and microbial competition, making field-scale quantification challenging (Cope et al., 2019). Theoretical and experimental work synthesizes these mechanisms, necromass stabilization, MAOC formation, and the suppression of free-living decomposers (the “Gadgil effect”)—into a model predicting a substantially larger soil carbon pool in ECM-dominated forests compared to those dominated by other mycorrhizal types (Averill and Hawkes, 2016). This model provides a powerful mechanistic framework. However, its validation across diverse forest ecosystems is ongoing, as the strength of decomposition suppression and the longevity of fungal necromass are context-dependent and influenced by factors such as soil nitrogen availability and the community composition of both ECM fungi and saprotrophic competitors (Averill and Hawkes, 2016). ECM-dominant forests exhibit higher carbon sequestration rates due to enhanced soil aggregation and reduced decomposition of organic matter (Yan et al., 2024). Temperate and boreal forests rely heavily on ECM mushrooms for maintaining soil carbon. These fungi release extracellular enzymes that regulate the degradation of complex organic compounds, enabling the storage of recalcitrant carbon and minimizing its cycling (Clemmensen et al., 2013). Experiments show that ECM mushrooms can account for up to 30% of forest soil carbon pools, highlighting their importance as contributors to climate change mitigation (Terrer et al., 2016).

Despite the evolutionary loss of many saprotrophic genes, some ECM mushrooms (e.g., *Paxillus involutus*) retain oxidative enzymatic activities to access organic nitrogen, particularly in nitrogen-limited boreal ecosystems. Using Fenton chemistry, *P. involutus* oxidizes soil organic matter (SOM) to release nitrogen, yielding smaller, polar metabolites that are adsorbed on mineral surfaces. This process not only facilitates nutrient uptake but also stabilizes carbon in the long term (Tunlid et al., 2022). Extramatrical mycelia (EMM) of ECM fungi are primary conduits of plant-carbon transfer into the soil. EMM biomass ranges from short-lived hyphae to perennial sclerotia, and its carbon sequestration contribution is controlled by turnover rates. Climate change has the potential to shift fungal communities and carbon allocation patterns, but the role of decomposers and grazers in these processes is poorly understood. Improved measurements are needed to quantify EMM turnover and its contribution to soil carbon storage (Cairney, 2012).

Other ECM fungi, for instance, *Cortinarius, Cenococcum*, and *Russula*, sequester soil carbon by modifying SOM rather than degrading it. *Cortinarius* secretes peroxidases to break down SOM in order to acquire nitrogen, while melanized *Cenococcum* hyphae are not broken down, allowing for long-term sequestration of carbon.

These different strategies highlight how fungal ecological processes are determined by both genetic traits and environmental factors (Bödeker et al., 2014). The lysis of ECM hyphae from *Russula* and *Boletus* enhances the development of MAOC, a recalcitrant pool of soil organic carbon. Nitrogen addition (25–50 kg N ha^−1^ year^−1^) can increase by 112%, stimulated by high biomass and microbial necromass and low SOC-degrading enzyme activity. Particularly, *Boletus*' resistant, melanized hyphae promote long-term carbon sequestration (Gao et al., 2024). Boreal forest carbon, representing a third of the world's soil carbon, is subject to decomposition under climate conditions. Activities like root tannin–fungal necromass binding decelerate decay by 30–50%, while melanized fungal residues (e.g., *Cortinarius*) are resistant to degradation. However, some ECM fungi (e.g., *Paxillus involutus*) break down mineral-bound carbon, challenging the presumption of its stability. Soil fauna play a role but require further investigation (Adamczyk, 2021). Fertilization effects on ECM fungi vary geographically. Nitrogen fertilization reduced ECM fungal growth and carbon deposition (300–1100 kg C ha^−1^ year^−1^) in control plots, corresponding to fungal biomass, at a Norway spruce stand.

Balanced nutrient applications in (N together with other nutrients) had less negative effects than nitrogen alone. Tree growth responses influenced these outcomes, highlighting the ongoing importance of ECM fungi for carbon sequestration (Wallander et al., 2011). Besides carbon stabilization, ECM fungi increase nutrient uptake (e.g., nitrogen and phosphorus) by the release of enzymes and mineral dissolution (e.g., apatite by oxalate and citrate). They invest 20–30% of photosynthesates into their hyphae, which also store carbon. Some ECM fungi degrade lignin and cellulose, thereby reducing environmental stressors, including drought and heavy metal toxicity. They promote plant-microbe interactions, i.e., symbiosis with *Burkholderia* and suppression of *Fusarium*, and increase seedling survival in degraded soils, particularly post-mining and post-fire soils (Itoo and Reshi, 2013). ECM Basidiomycetes, for example, *Pholiota* rapidly colonize fire-scarred soils in post-fire communities, creating networks that stabilize the substrate and allow early nutrient cycling and recovery (Claridge et al., 2009). Fungi like *Lactarius* and *Laccaria* rapidly mineralize ECM necromass (e.g., *Pisolithus microcarpus*), incorporating^13^C-enriched carbon within days, thereby emphasizing their role in the rapid cycling of soil carbon (Drigo et al., 2012). Experimental field evidence shows that ECM fungi can suppress soil respiration by 67% by competing with decomposer microbes for nitrogen. Basidiomycetes particularly, *Laccaria* and *Russula* are especially effective in such a role, suppressing microbial activity and promoting carbon sequestration (Averill and Hawkes, 2016). Nitrogen enrichment in the boreal forest alters fungal communities, reducing the occurrence of Basidiomycete ECM fungi (e.g., *Cortinarius)* that facilitate SOM decomposition. This shift is associated with reduced decomposition and enhanced carbon sequestration.

While total fungal biomass may rise by Ascomycete growth, the decline of Basidiomycetes reflects a biodiversity–function trade-off (Hannula and Morriën, 2022; Jörgensen et al., 2022). Contrary to earlier assumptions, ECM Basidiomycetes for instance *Piloderma, Amphinema*, and *Wilcoxina* compete with saprotrophs to break down SOM by hydrolytic and oxidative enzymes. Both enzymatic processes play key roles in labile and recalcitrant carbon cycling in sub-boreal ecosystems (Phillips et al., 2014). Succession in Swedish boreal forests demonstrate the changes in the functions of mycorrhizal fungi. In the early stages, cord-forming ECM Basidiomycetes like *Cortinarius* and *Suillus* dominate, facilitating rapid carbon and nitrogen cycling. Over time they are replaced by ericoid mycorrhizal Ascomycetes bearing melanized, decay-resistant hyphae that contribute to the buildup of SOM, with evidence for the dynamic and transition modes of fungal contribution to carbon in soil (Clemmensen et al., 2015). Morphologically, the ECM fungi display a sheath (mantle), a Hartig net, and extramatrical mycelia that extend into the soil. Common genera, such as *Russula, Lactarius*, and *Boletus*, are widespread across most forest ecosystems, especially in tropical, temperate, and boreal ecosystems. They contribute to nutrient uptake, store 50% of carbon fixed by vegetation, and enhance plant resistance to stresses (Sharma et al., 2020). ECM fungi also serve as bioindicators of heavy metal contamination, providing information on environmental pollution and soil quality (Ediriweera et al., 2022). Their capacity to bioaccumulate trace elements makes them useful for ecological monitoring.

Edible ECM species, including *Tuber* and *Cantharellus*, bioaccumulate and excrete carbon molecules, hence influencing the flow of nutrients and strengthening microbial interactions in forest ecosystems (Rangel-Castro et al., 2002; Boroujeni and Hemmatinezhad, 2015). Fungi are also involved in maintaining soil mesofauna, which in turn affects fungal growth and decomposition processes (Hernández-Santiago et al., 2020). Mycorrhizal fungi for example *Boletus edulis* sequester organic carbon and increase ecosystem resilience in forest ecosystems (Ogwu et al., 2025a; Phillips, 2017). Such interactions with macro- and microfauna and their role in biological weathering processes and long-term carbon dynamics account for their role in ecosystem stability maintenance (Lang, 2020; Liu et al., 2021).

#### 2.1.2 Arbuscular mycorrhizal fungi (AMF) and carbon sequestration in agricultural systems

While this review focuses on the role of mushrooms, the macroscopic fruiting bodies of primarily Basidiomycota and Ascomycota, it is critical to consider the broader functional ecology of fungi in the carbon cycle. Arbuscular mycorrhizal fungi (AMF; Glomeromycota) do not produce mushrooms, but their symbiotic principles and profound impact on soil carbon sequestration provide an essential contrast to ectomycorrhizal (ECM) systems and are indispensable for a holistic understanding of fungal-mediated carbon management, particularly in agricultural landscapes. Arbuscular mycorrhizal fungi (AMF), including dominant species like *Glomus intraradices* and *Rhizophagus irregularis*, are key agents of carbon sequestration in agro-landscapes with mechanisms distinct from their ECM counterparts (Shukla et al., 2025; Busso and Busso, 2022). Although ECM fungi form symbiotic relationships primarily with forest tree species (Smith et al., 2011), AMF associate with approximately 80% of herbaceous arable crops (Fall et al., 2022) and are thus central partners in agri-food production systems. This specialization enables AMF to enhance crop resistance to abiotic stresses like drought, soil erosion, and nutrient deficiency (Usman et al., 2021; Sangwan et al., 2023) while also promoting long-term carbon sequestration through various biochemical pathways.

The functional differentiation between ECM fungi and AMF is evident in their carbon stabilization mechanisms. AMF excrete glomalin, a glycoprotein not produced in ECM systems, which complexes with soil particles to create stable aggregates lasting 6–42 years (Rillig et al., 2001; Zhu and Miller, 2003). Glomalin-associated soil proteins account for 4–5% of agricultural soil carbon (Singh et al., 2013), creating a substantial carbon pool that often exceeds microbial biomass inputs. Their extensive hyphal networks are estimated to channel 15–20% more photosynthate belowground than non-mycorrhizal roots, based on recent isotopic labeling studies (Li et al., 2025). This substantial belowground carbon allocation is a key trait supporting the hypothesis that AMF are significant agents for carbon sequestration in agricultural soils (Rillig et al., 2015; Yang et al., 2022). It is important to note that this percentage, like its ECM counterpart, is influenced by plant host, soil phosphorus status, and agricultural management (e.g., tillage). Furthermore, the net sequestration benefit depends on the fate of this hyphal carbon, whether it is rapidly consumed by soil fauna and microbes or stabilized in aggregates via glomalin—a variable not fully captured in short-term labeling experiments (Rillig et al., 2015; Yang et al., 2022).

For agricultural practitioners, these biological processes have management implications. Minimizing soil disturbance through conservation tillage systems increases AMF colonization and soil organic carbon by 15–20% compared to conventional tillage (Phillips et al., 2014; Yang et al., 2024). Strategic reduction of phosphorus inputs is also crucial, since excessive fertilizers inhibit AMF activity, while balanced application can optimize the symbiosis (Ryan and Kirkegaard, 2012). Experimental field trials indicate that AMF inoculation in degraded land can enhance carbon sequestration by 30–50% over three years (Yin et al., 2016), whereas integrated crop-mushroom rotation systems increase fungal necromass input by 25% (Dou et al., 2025). Collectively, these practices reduce CO_2_ emissions from AMF-dense agricultural ecosystems (Cobb et al., 2016; Mason et al., 2025).

The rapid turnover of AMF hyphae reveals an interesting ecological mechanism: while the hyphae themselves are ephemeral, their chitinous detritus, together with ongoing glomalin deposition, stabilizes aggregates over the long term (Zhu and Miller, 2003; Singh et al., 2013). New isotopic labeling experiments suggest that AMF inoculation increases occluded particulate organic carbon (oPOC) and MAOC by 15.6% and 7.1%, respectively, in agricultural soil (Li et al., 2025). Practitioners should be aware of potential trade-offs, such as reduced bacterial diversity due to competition for nitrogen (Li et al., 2025). These findings highlight the dual benefit of AMF-mediated carbon sequestration: enhancing crop yield while contributing to climate change mitigation (Bender et al., 2016; Devi et al., 2021), particularly when integrated into conservation agriculture practices (Prasad, 2020; Oruru and Njeru, 2016).

The body of evidence confirms that AMF are a core component of sustainable agriculture (Sangwan et al., 2023; Tao and Liu, 2024) and can be harnessed by farmers to improve soil health and contribute to global carbon solutions. By understanding the distinct ecological roles of ECM fungi and the practical benefits of AMF outlined above, agricultural stakeholders can make informed decisions about adopting AMF-conducive practices in various production systems.

#### 2.1.3 Saprotrophic decomposition and carbon turnover

Through enzymes like laccases and peroxidases, saprotrophic fungi break down lignocellulose and can contribute to the formation of stable humic compounds. Some studies suggest that this fungal-mediated transformation can result in a lower net CO_2_ release per unit of decomposed material than pathways dominated by bacterial decomposition (Martínez-García et al., 2017; Baldrian et al., 2023). This comparative efficiency is a central but nuanced concept. It is highly dependent on fungal species (e.g., “humus-producing” vs. “fast-decomposing” strategies), substrate quality, and environmental conditions like temperature and moisture. The generalization that fungi are universally more efficient than bacteria in stabilizing carbon is an oversimplification; the outcome is governed by the specific functional traits of the microbial community present (Baldrian et al., 2023; Martínez-García et al., 2017). This biochemical capability enables many saprotrophic fungi to channel a large proportion of decomposition-derived carbon into long-lasting soil reservoirs, where fungal necromass and enzyme-bound organic substrates significantly contribute to recalcitrant carbon pools that can persist for centuries (Cotrufo et al., 2013; Fu et al., 2025).

The climate change mitigation potential of saprotrophic fungi is highly variable and depends on species-specific decomposition strategies. Fast-decomposing fungi, such as *Coprinopsis* species, rapidly mineralize organic carbon and can release a large proportion (up to 70%) as CO_2_. In contrast, humus-producing species like *Ganoderma* exhibit higher carbon conversion efficiencies, channeling over half of the decomposed carbon into stable soil aggregates (Shao et al., 2023; Schneider et al., 2012). This functional specialization involves important trade-offs that are further influenced by climate, as cold climates favor darker-pigmented saprotrophs that degrade organic matter more slowly but store carbon more efficiently than their tropical counterparts (Krah et al., 2019). These differences suggest that land management can influence the fate of carbon mediated by fungi, as demonstrated by a 22% increase in mineral-associated organic carbon following targeted inoculation of farm residue with *Stropharia rugosoannulata* (Hao et al., 2024), or a 35% enhancement in microbial carbon use efficiency with spent mushroom substrate (SMS) amendments (Chen L. et al., 2024).

However, saprotrophic fungal carbon sequestration is increasingly susceptible to climate change. For example, soil erosion has led to 40–60% decreases in productivity of ecologically important decomposers like *Morchella* species (Zhang Y. et al., 2023), and nitrogen deposition alters the fungal community in ways that favor fewer effective decomposers (Sommer et al., 2021). These constraints necessitate the maintenance of diverse saprotrophic communities, as traditional agriculture systems demonstrate that food security and enhanced carbon sequestration are linked to maintaining fungal diversity (Wendiro et al., 2019; Liu et al., 2021). The complex role of saprotrophic fungi in soil carbon cycling positions them as essential yet challenging agents in climate change mitigation, requiring careful management to balance their decomposition activity with their carbon sequestration potential across diverse ecosystems. The functional roles, mechanisms, and ecological impacts of key fungal groups in terrestrial carbon cycling are synthesized in Table 1.

**Table 1:** Functional classification of fungi in terrestrial carbon cycling and stabilization.

**Functional guild and example genera/species**	**Primary ecosystem role**	**Key mechanisms for carbon mitigation**	**Typical evidence and scale**	**Estimated impact/key findings**	**References**
Ectomycorrhizal (ECM) symbionts (*Amanita, Boletus, Cortinarius, Laccaria, Pisolithus, Russula*)	Obligate symbionts with tree roots in boreal, temperate, and some tropical forests.	(1) Necromass stabilization: Melanized hyphae (e.g., *Cenococcum*) resist decay. (2) MAOC formation: hyphal lysis products bind to soil minerals. (3) Gadgil effect: suppresses SOM mineralization by competing with decomposers	Field isotopic tracing (^13^C, ^15^N); forest comparison plots; controlled pot experiments	It can account for ~30% of forest soil C pools. ECM-dominated forests may store up to 70% more belowground C. improves soil aggregation	Wallander et al., 2011; Clemmensen et al., 2013, 2015; Bödeker et al., 2014; Phillips et al., 2014; Averill and Hawkes, 2016; Terrer et al., 2016; Cope et al., 2019; Gao et al., 2024
Arbuscular mycorrhizal fungi (AMF) (*Glomus/Rhizophagus, Funneliformis*)	Symbionts of ~80% of herbaceous plants; dominant in grasslands and agricultural systems	(1) Glomalin production (GRSP): persistent aggregate stabilizer. (2) Hyphal C transfer: channels plant C into soil. (3) Aggregate formation: physical binding of soil particles	Long-term agricultural trials; GRSP extraction; ^13^C labeling in mesocosms	GRSP = 4–5% of agricultural soil C. AMF inoculation increases C sequestration by 30–50% over 3 years. SOC increases 15–20% under conservation tillage	Rillig et al., 2001, 2015; Zhu and Miller, 2003; Singh et al., 2013; Bender et al., 2016; Fall et al., 2022; Yang et al., 2022, 2024; Li et al., 2025
Saprotrophic decomposers (humifying) (*Ganoderma lucidum, Stropharia rugosoannulata)*	Decompose lignocellulosic residues in forests and agroecosystems	Humification and necromass input: high C-use efficiency; produces humic compounds and stable fungal necromass entering slow SOC pools	Solid-state fermentation; field trials with soil amendments; enzyme assays	Increases mineral-associated OC by ~22%. SMS amendments enhance microbial activity and SOC storage	Martínez-García et al., 2017; Baldrian et al., 2023; Shao et al., 2023; Chen L. et al., 2024; Hao et al., 2024; Dou et al., 2025
Saprotrophic decomposers (mineralizing) (*Coprinopsis* spp.)	Rapid turnover of labile organic matter	Rapid Mineralization: Fast growth and high respiration mineralize organic C to CO_2_	Laboratory microcosms; short-term decomposition studies	Can mineralize >70% of substrate C to CO_2_	Schneider et al., 2012; Shao et al., 2023
Integrated bioeconomy agents (cultivated *Pleurotus* spp*., Lentinula edodes*)	Cultivated on agro-industrial wastes for food, feed, and soil amendment.	Cascade valorization: (1) C stored in edible fungal biomass. (2) Post-harvest SMS adds stable organic matter and improves soil structure.	Pilot-scale biorefineries; LCA studies; field trials using SMS.	SMS application can sequester 0.5–2.0 t CO_2_-eq ha^−1^ year^−1^. converts waste to protein, bioenergy, and soil C	Liu et al., 2021; Kulshreshtha et al., 2014; Zhang W. et al., 2023; Ramamoorthy et al., 2023; Dou et al., 2025
General saprotrophic community and indicators (wild saprotrophs*, Agaricus bisporus, Morchella*)	Diverse decomposers respond to soil conditions and disturbances	C-Nexus and bioindication: Influence C accumulation; δ ^13^C and δ^15^N signatures indicate SOM cycling and environmental stress	Field surveys; isotopic analysis of sporocarps; cultivation studies	N deposition alters community composition and decomposition rates. Morel productivity can decline by 40–60% due to soil erosion	Komárek et al., 2007; Kyaschenko et al., 2017; Krah et al., 2019; Sommer et al., 2021; Zhang Y. et al., 2023

### 2.2 Critical evaluation of methodologies in fungal carbon cycling research

While the studies summarized in Tables 1–3 present a compelling case for fungi as major agents of carbon sequestration, a critical evaluation of the methodologies underlying these studies reveals significant limitations and uncertainties that constrain our ability to extrapolate these findings to predictive, field-scale climate solutions.

#### 2.2.1 Laboratory vs. field discrepancies

Foundational claims, such as the suppression of soil respiration by ECM fungi by up to 67%, originate from highly controlled greenhouse or growth chamber experiments using simplified tree-fungus pairs (e.g., *Pinus taeda* with *Laccaria bicolor*) in sterile or semi-sterile soil (Averill and Hawkes, 2016). These microcosm studies effectively isolate mechanisms but systematically exclude key biotic interactions, particularly grazing by soil micro- and mesofauna, which can rapidly consume fungal hyphae and necromass, accelerating carbon turnover in natural systems (Pollierer et al., 2012). Consequently, the net carbon-stabilization effect observed in the lab may be significantly overestimated under complex field conditions.

#### 2.2.2 Scale and isotopic tracing

More robust evidence comes from field-based isotopic tracing (e.g., Clemmensen et al., 2013). However, these studies are often limited to specific ecosystems (e.g., boreal forests) and short-term (< 5 years) pulses. The long-term fate of fungal-derived carbon—whether it remains in stable mineral-associated pools or is eventually remineralized—remains poorly constrained. Furthermore, methodological differences in measuring fungal biomass (ergosterol vs. sequencing vs. hyphal ingrowth bags) and its turnover make cross-study comparisons difficult and meta-analyses challenging.

#### 2.2.3 Environmental context dependency

The functional outcomes of fungal communities are highly context-dependent. For instance, the “Gadgil effect” (decomposition suppression by ECM fungi) appears strongest in nitrogen-limited systems (Wu et al., 2022), while nitrogen deposition can shift communities toward less efficient decomposers (Jörgensen et al., 2022). Most studies examine single factors (e.g., N addition, warming). Multi-factorial experiments that combine warming, drought, and altered precipitation—mimicking real-world climate change are rare but essential, as they often reveal non-additive effects that challenge predictions from single-stressor studies (Fernandez et al., 2017).

#### 2.2.4 Inconsistencies in saprotroph function

For saprotrophic fungi, the dichotomy between “fast” (high CO_2_ release) and “slow” (high humification) decomposers is useful but often oversimplified. In reality, decomposition strategies exist on a spectrum and are influenced by substrate quality and climate. The common practice of inoculating single, often commercial, fungal strains (e.g., *Pleurotus ostreatus*) onto agricultural wastes provides valuable data on bioconversion efficiency but tells us little about how native, complex saprotrophic communities in forests or grasslands will respond to climate change. This represents a critical gap between applied biotechnology and ecosystem ecology.

### 2.3 Synthesis and future research priorities: toward predictive understanding

The dual role of fungi, as decomposers releasing CO_2_ and as symbionts stabilizing carbon, creates a complex dynamic central to the terrestrial carbon balance. A critical knowledge gap is our inability to quantitatively predict the net carbon outcome of fungal community shifts under climate change, fertilization, or land-use alteration. Specifically, the trade-offs between saprotrophic decomposition rates and mycorrhizal carbon allocation to slow-cycling pools remain poorly constrained. Future research must employ isotopic tracing (e.g., ^13^C, ^15^N) in tandem with molecular characterization of fungal necromass to move from correlative studies to a mechanistic, predictive understanding of how specific fungal traits and interactions govern long-term soil carbon storage.

## 3 Core mechanism II: enzymatic degradation and detoxification of pollutants

The climate mitigation potential of mushrooms is powerfully demonstrated by their extracellular enzymatic systems, which detoxify environments by decomposing harmful pollutants and restoring contaminated ecosystems. They degrade organic contaminants, heavy metals, and anthropogenic chemicals using extracellular enzymes and biosorption, making them vital tools for bioremediation in various ecosystems (Singh et al., 2021). Their ability to degrade recalcitrant pollutants underscores their potential in mitigating industrial, agricultural, and petroleum pollution.

### 3.1 Mycoremediation of pollutants

**Figure 2:**
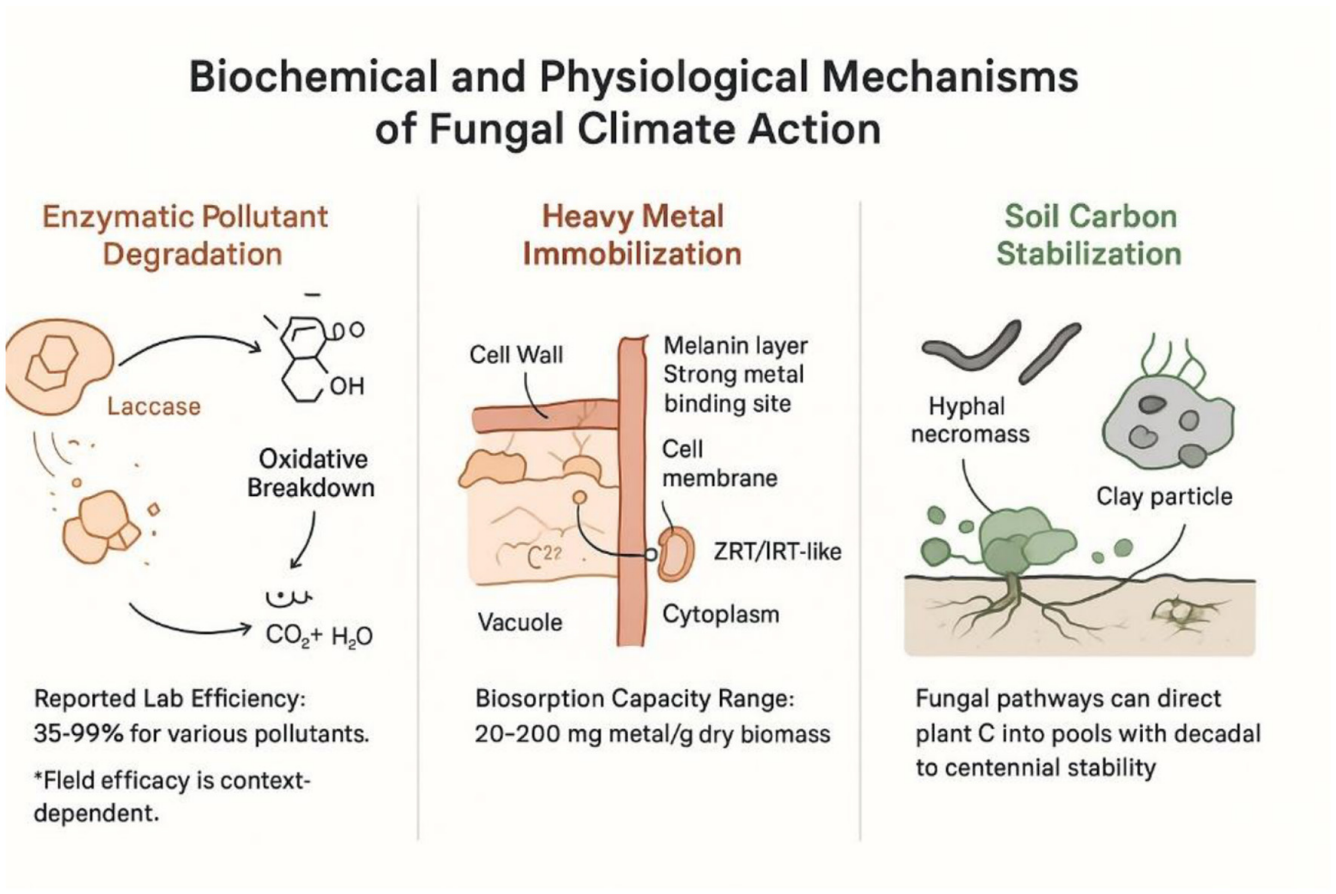
Biochemical and physiological mechanisms underpinning fungal climate mitigation.

Mycoremediation is a bioremediation approach that harnesses the enzymatic systems of fungi to transform or sequester pollutants. The underlying biochemical and physiological mechanisms, encompassing enzymatic degradation, metal biosorption, and carbon stabilization, are summarized in Figure 2.

In controlled laboratory studies, numerous mushroom species demonstrate remarkable potential to degrade complex hydrocarbons, dyes, and other persistent organic pollutants, thereby establishing the biochemical basis of the technique (Stamets, 2019; Gugel et al., 2024; Maddala et al., 2025). However, a persistent and significant gap exists between these promising *in vitro* results and reliable, predictable field-scale applications. This gap stems from the complex interplay of factors in real-world environments, including microbial competition, heterogeneous contaminant mixtures, and suboptimal physical conditions, which are often absent in standardized lab assays (Stamets, 2019; Gugel et al., 2024; Maddala et al., 2025). Besides being a nutritious food source, mushrooms are also effective agents in mycoremediation, capable of degrading pollutants including plastics and heavy metals through biodegradation and biosorption (Ikhimalo and Ugbenyen, 2023; Sharma, 2025). However, safety tests are necessary, as mushrooms grown on polluted substrates can accumulate toxicants, rendering them unsafe for consumption (Kulshreshtha et al., 2014; Malik et al., 2021; Qin et al., 2024).

Mycoremediation is generally a non-toxic process that minimizes the production of toxic byproducts (Ab Rhaman et al., 2022). Medicinal and food mushrooms play a significant role in biodegradation, biosorption, and bioconversion during decontamination (Kulshreshtha et al., 2014; Malik et al., 2021; Dinakarkumar et al., 2024). They are effective decomposers of lignin, cellulose, and hemicellulose, and are also capable of accumulating and storing toxic substances, such as heavy metals (Bucurica et al., 2024; Ogwu et al., 2025b). With the exception of *Serpula himantioides*, genera such as *Agaricus, Amanita, Boletus, Cortinarius, Leccinum, Suillus*, and *Phellinus* have been found to be efficient in mobilizing and detoxifying soil pollutants, and some can even competitively inhibit bacteria during the degradation of xenobiotic compounds and heavy metals (Malik et al., 2021; Gupta et al., 2024). Inoculation of soils with native mushroom strains or their mycelial inoculum can significantly increase the removal of pollutants (Medaura et al., 2021). Biodegradation refers to the microbial breakdown of organic substances through processes including biodeterioration, fragmentation, and assimilation (Fouad et al., 2022; Ikhimalo and Ugbenyen, 2023; Crescent et al., 2025). Fungal genera such as *Pleurotus* and *Ganoderma* produce extracellular enzymes capable of breaking down complex pollutants, including polycyclic aromatic hydrocarbons (PAHs), plastics, synthetic dyes, crude oil, and radioactive waste (Akpasi et al., 2023; Ali et al., 2025; Fonseka et al., 2025). Their extensive hyphal systems enable the adsorption and uptake of toxic metals (Dinakarkumar et al., 2024).

Bioconversion involves the transformation of lignocellulose from industrial and agricultural wastes into useful bioproducts by fungal metabolism. White-rot fungi, such as *Pleurotus* and *Agaricus*, produce ligninolytic enzymes, particularly laccases and peroxidases, to degrade lignin and a vast array of xenobiotics (Gałazka et al., 2025; Ibarra-Rondón et al., 2025). In addition, cellulases and related enzymes break down cellulose, facilitating the production of enzymes, proteins, and secondary metabolites (Goswami et al., 2025). Mushroom cultivation of species such as *Lentinula edodes, Pleurotus eous*, and *Macrocybe titans* on agro-waste substrates converts biomass into useful commercial products (Rani et al., 2008; Kulshreshtha et al., 2014; Kumar P. et al., 2022). Biosorption is the spontaneous adsorption of metal ions and contaminants by fungal biomass from liquid systems. *Lentinus tuberregium, Pleurotus platypus, Calocybe indica*, and *Agaricus bisporus* have demonstrated great potential for heavy metal sequestration, particularly of Cu, Zn, Fe, Cd, Pb, and Ni (Kumar V. V. et al., 2022; Beig and Shah, 2023). Metal distribution within mushrooms is not uniform; concentrations are typically higher in caps than in stipes and vary with substrate composition, mycelium maturity, and developmental stage (Andronikov et al., 2023; Soceanu et al., 2024). The efficacy of key fungal species against major classes of organic pollutants is synthesized in Table 2.

**Table 2:** Efficacy of selected mushrooms in the mycoremediation of organic pollutants.

**Fungal species**	**Pollutant class**	**Specific pollutant**	**Key findings and reported efficacy**	**Primary mechanism/ enzymes**	**References**
*Pleurotus ostreatus*	Petroleum hydrocarbons and PAHs	TPH, phenanthrene, Pyrene, benzo[a]pyrene	Achieves 85–95% PAH degradation in 30–60 days; also effective on crude oil contamination	Laccase, MnP, LiP (ligninolytic enzymes)	Sack and Günther, 1993; Eggen and Majcherczyk, 1998; Arun et al., 2008; Okparanma et al., 2011; da Luz et al., 2013; Kosnar et al., 2018; Elhusseiny et al., 2019; Dickson et al., 2020; Mayans et al., 2024
	Chlorinated pesticides	Aldrin, dieldrin, heptachlor, DDT	100% Aldrin removal; 89% Heptachlor removal at 50 mg/kg; DDT degraded with microbial partners	Oxidative dechlorination; side-chain oxidation	Purnomo et al., 2014, 2017, 2019
	Industrial dyes	Direct Red 5B, Direct Blue 22, and Coralene dyes	88–98% dye decolorization	Laccase, peroxidases	Madhavi and Lele, 2009; Kunjadia et al., 2016; Alhujaily et al., 2020
	PCBs	Delor 103, Aroclor mixtures	94.1–99.6% PCB removal in soil/liquid assays	Broad-specificity peroxidases	Zeddel et al., 1993; Cvančarová et al., 2012; Siracusaa et al., 2017
	Pharmaceuticals	Sulfonamides, diclofenac, lamotrigine	Removes 43–91% depending on compound and matrix	Extracellular oxidative enzymes	Lucas et al., 2016; Chefetz et al., 2019; Hultberg et al., 2020
	Plasticizers	DBP, BBP	High removal efficiency; enzymatic cleavage of ester bonds	Esterases, oxygenases	Ahuactzin-Pérez et al., 2018; Chang et al., 2021
*Trametes versicolor*	PAHs and phenolics	Phenanthrene, BaP, PCP	>60–91% degradation of high-MW PAHs and PCP	Laccase, peroxidase systems	Wesenberg et al., 2003; Nguyen et al., 2014; Vipotnik et al., 2022
	Industrial dyes and textile effluent	Azo and anthraquinone dyes	Up to 98% decolorization; effective for mixed effluents.	Laccase-mediated oxidative breakdown	Amaral et al., 2004; Sivasakthivelan, 2013
	Endocrine disruptors	BPA, 4-tert-octylphenol	>90% degradation with reduced toxicity	Laccase (often with mediators)	Nguyen et al., 2014; Liu H. Y. et al., 2019
*Phanerochaete chrysosporium*	PAHs	Acenaphthene, phenanthrene, pyrene	Complete (100%) degradation of selected PAHs within 7 days at 100 mg/L with MnSO_4_	LiP, MnP	Leonardi et al., 2007; Abo-State et al., 2021
	PCBs and halogenated compounds	Aroclor 1242, 1254, 1260	Effective degradation in liquid cultures; weaker in non-sterile soils	LiP/MnP	Bumpus et al., 1985; Eaton, 1985; Chun et al., 2019
	Dyes and pesticides	Azo dyes; 2,4-D	Broad xenobiotic degradation capacity	LiP, MnP	Bumpus, 2004; Abatenh et al., 2017
*Ganoderma lucidum*	PAHs	Phenanthrene, pyrene	Up to 99.6% degradation within 30 days	Ligninolytic enzymes	Agrawal et al., 2018
	Pharmaceuticals	Carbamazepine, diclofenac	13–34% removal from water	Biosorption + enzymatic transformation	Lucas et al., 2016
*Irpex lacteus*	PCBs and chlorinated compounds	PCB mixtures, chlorobenzaldehydes	39% PCB degradation in soil; transforms chlorinated benzoates	MnP; extracellular enzymes	Stella et al., 2017; Šrédlová et al., 2021
*Lentinula edodes*	Chlorophenols and PCBs	2,4-DCP, PCB mixtures	Degradation enhanced by mediators (e.g., vanillin)	Laccase, peroxidase	Ruiz-Aguilar et al., 2002; Tsujiyama et al., 2013
*Ceriporia lacerata*	PAHs	Pyrene	55.5–90.5% degradation in 8 days with glucose addition	Ligninolytic enzyme activity	Al-Hawash et al., 2022
*Coriolus versicolor*	PAHs, dyes	Various dyes and lignin-related pollutants	Up to 98% dye decolorization; strong PAH degradation	Ligninolytic enzymes	Jang et al., 2009; Sivasakthivelan, 2013
*Lentinus tigrinus*	PCBs, chlorobenzoates	PCBs, CBAs	Effective PCB and chlorobenzoate degradation.	Laccases, peroxidases	Stella et al., 2012, 2013
*Peniophora incarnata*	PAHs	Phenanthrene, anthracene	91% removal of phenanthrene and 71% removal of anthracene	Oxidative enzymes	Tan, 2023
*Phlebia acerina*	PAHs	Benzo[a]pyrene	57.7% degradation in 32 days	Ligninolytic enzymes	Zhang W. et al., 2023
*Pleurotus dryinus/pulmonarius*	PAHs and TPH	Crude oil PAHs and TPH	40–97% degradation depending on substrate	Oxidative enzymes	Olusola and Anslem, 2010; Rathankumar et al., 2020

#### 3.1.1 Degradation of petroleum hydrocarbons by mushrooms

**Figure 3:**
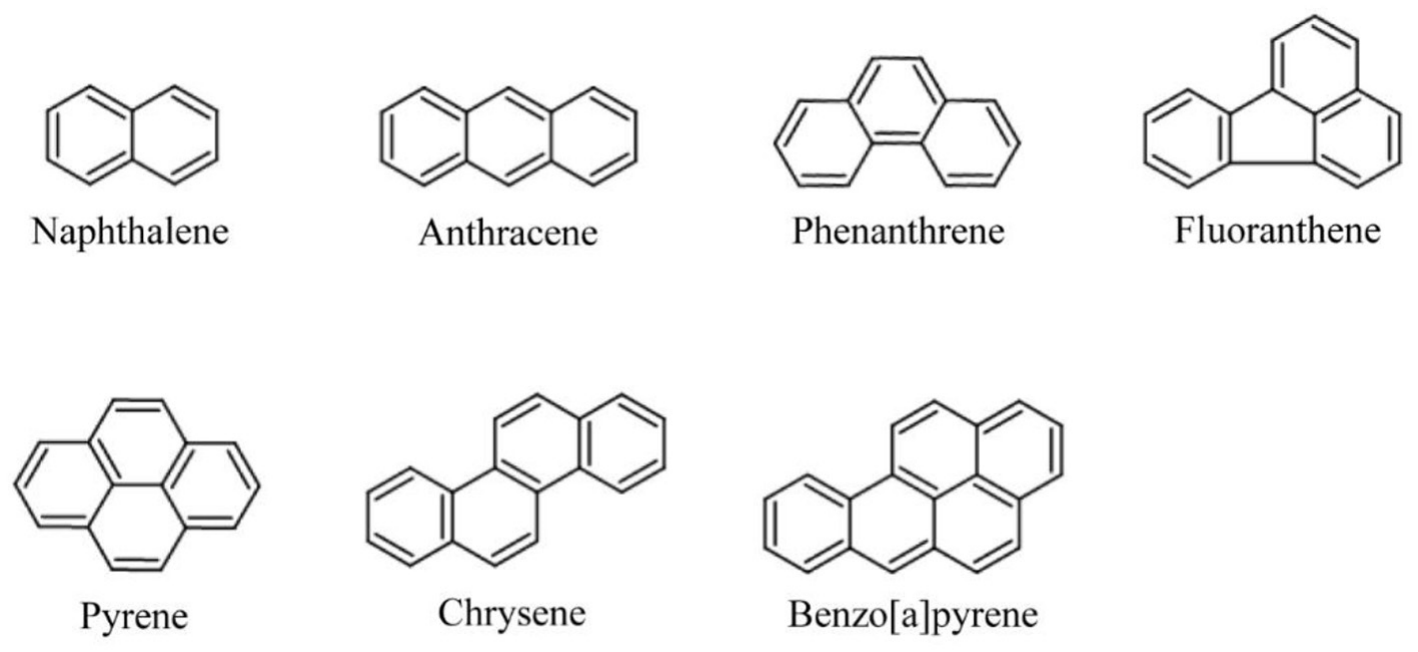
Molecular structures of representative polycyclic aromatic hydrocarbons (PAHs) targeted in mycoremediation (ChemDraw Pro 8.0). The depicted PAHs, including naphthalene (2-ring), phenanthrene and anthracene (3-ring isomers), and pyrene (4-ring), are common, persistent constituents of petroleum hydrocarbons and industrial pollution.

Petroleum contamination and oil spills are serious environmental problems that threaten aquatic and terrestrial life. Saprotrophic mushrooms like *P. ostreatus* are highly efficient at degrading petroleum hydrocarbons, including polycyclic aromatic hydrocarbons (PAHs) and alkanes (Singh et al., 2021; Mayans et al., 2024; Crescent et al., 2025; Figure 3).

The ligninolytic enzymes, such as laccases and manganese peroxidases, break down recalcitrant hydrocarbons into less toxic products that are more readily degraded by natural biogeochemical processes (Kuhad et al., 2011; Kumar V. V. et al., 2022; Ramamurthy et al., 2024). Substrates inoculated with *Pleurotus* species effectively degrade hydrocarbons and can thus be utilized for the bioremediation of oil spills (Bhatt et al., 2023; Okpa et al., 2024).

Organic xenobiotic pollutants (XOCs), such as petroleum hydrocarbons, PAHs, dyes, and halogenated compounds, are persistent, non-degradable man-made pollutants with significant environmental and health impacts. Emitted into the environment from industry, agriculture, and the combustion of fossil fuels in large quantities, these pollutants persist in soil and bioaccumulate in food webs due to their hydrophobicity (Miglani et al., 2022; Khushboo et al., 2024; Tarrad et al., 2024). Fungal biodegradation of contaminants through mycoremediation is a cost-effective and environmentally friendly remediation strategy. White-rot fungi such as *S. rugosoannulata* and *P. ostreatus* can secrete extracellular ligninolytic enzymes such as manganese peroxidase, lignin peroxidase (LiP), and laccase to degrade a wide range of complex organic contaminants (Zhuo and Fan, 2021; Pozdnyakova et al., 2022). For example, *P. ostreatus* can degrade up to 95% of PAHs in contaminated soil. Meanwhile, *S. rugosoannulata* exhibits superior biodegradation potential for high-molecular-weight PAHs, such as anthracene and benzo[a]pyrene (Steffen et al., 2017). Fungi also degrade halogenated xenobiotics, particularly TCE, PCBs, and dioxins, by reducing their toxicity, mutagenicity, and environmental persistence (Aguilar et al., 2024). Overall, fungi mitigate the environmental threat of XOCs through mycoenzymatic degradation, mycosorption, and mycoconversion. The following data (Table 3) compiles reported degradation efficiencies from a wide array of studies, often showcasing high removal percentages. The reader is cautioned that these values predominantly reflect performance under optimized, small-scale laboratory conditions (e.g., sterile soil, single contaminants, ideal moisture/temperature). Key challenges of field application, such as microbial competition, contaminant mixture toxicity, and nutrient limitations, are systematically absent from these experimental setups, leading to an “optimization bias” in the literature. With this critical context in mind, Table 3 provides a catalog of fungal capabilities and the enzymatic foundations for mycoremediation.

**Table 3:** Mycoremediation potential of selected mushroom species in degrading petroleum hydrocarbons and related pollutants.

**Species**	**Pollutant degraded**	**Key findings**	**References**
*Agaricus bisporus*	PAHs	Effective in degrading condensed PAHs via bioaugmentation	García-Delgado et al., 2015
*Ceriporia lacerata*	Pyrene	Removed 55.5% in 8 days; increased to 90.5% with glucose	Al-Hawash et al., 2022
*Coriolus versicolor*	PAHs	Degrades lignin and PAHs via ligninolytic enzymes	Libra et al., 2003; Jang et al., 2009
*Coriolopsis byrsina*	Acetic acid anhydride	Effective degradation activity	Agrawal et al., 2021
*Fomes* sp.	Anthracene	Converted 100 μM into 9,10-anthraquinone	Godoy et al., 2016
*Ganoderma lucidum*	Phenanthrene, Pyrene	99.65% and 99.58% degradation in 30 days	Agrawal et al., 2018
	Carbamazepine, diclofenac, Iopromide, venlafaxine	Degraded: carbamazepine (13%), diclofenac (34%), Iopromide (18%), Venlafaxine (11%)	Lucas et al., 2016
*Irpex lacteus*	Polychlorinated biphenyls	39% PCB was degraded; Intracellular and extracellular enzymes played a vital role	Stella et al., 2017
*Lentinus tigrinus*	PCB (polychlorinated biphenyl)	Degrades PCBs and CBAs via laccases/peroxidases	Stella et al., 2012
*L. subnudus*	Crude oil	Effectively mineralized TPH in crude oil-contaminated soils. Biodegradation peaked at 20% oil concentration (3 months) and 40% (6 months). Increased organic matter, carbon, and nutrients, except potassium	Adenipekun and Fasidi, 2005; Adenipekun and Isikhuemhen, 2008
*Leucoagaricus gongylophorus*	Anthracene	Effective degradation via ligninolytic enzymes	Ike et al., 2019
*Marasmiellus* sp.	Pyrene	Achieved 100% degradation within 48 hours under saline conditions	Vieira et al., 2018
*Peniophora incarnata*	Phenanthrene, anthracene, benzo[a]pyrene	Removed: 91%, 71%, and 35% respectively	Tan, 2023
*Phanerochaete chrysosporium*	Acenaphthene, Phenanthrene, Pyrene	100% degradation at 100 mg/L in 7 days with MnSO_4_	Leonardi et al., 2007; Abo-State et al., 2021
*Phlebia acerina*	Benzo[a]pyrene	57.7% degraded in 32 days; more effective than *P. chrysosporium*	Zhang W. et al., 2023
*Pleurotus florida*	TPHs	55% of total petroleum hydrocarbons (TPHs) were degraded within 30 days due to the significant enzymatic activity of laccase and tyrosinase	Roshandel et al., 2021
*P. dryinus*	Phenanthrene (Phe), benzo[a]pyrene (BaP)	Phe: 97.43%; BaP: 54.45% degraded at 50 mg/kg	Rathankumar et al., 2020
*P. eryngii*	Aromatic hydrocarbons, DDT	Transformed into less toxic compounds, DDT degraded by 43%	Hadibarata et al., 2013; Purnomo et al., 2019
*P. ostreatus*	PAHs	36% degraded at 0.1 mg/kg	Sack and Günther, 1993; Eggen and Majcherczyk, 1998; Okparanma et al., 2011; Kosnar et al., 2018; Elhusseiny et al., 2019
	Anthracene, fluoranthene, benzo(a) pyrene	Degrade high-molecular-weight PAHs under solid-state fermentation conditions	Bojan et al., 1999; Bhattacharya et al., 2012
	Aldrin, Dieldrin	Aldrin: 100%, Dieldrin: 18% degraded at 50 mg/kg.	Purnomo et al., 2017
	Heptachlor, heptachlor epoxide	89% and 32% degradation at 50 mg/kg	Purnomo et al., 2014
	Polychlorinated biphenyls (PCBs)	94.1% degradation at 9.28 mg/kg	Siracusaa et al., 2017
	Acenaphthene, PAHs, plastics	Acenaphthene: 20.6% degraded; degraded plastic as growth substrate; 90% PAH degradation in 20 days	Arun et al., 2008; da Luz et al., 2013; Efenudu, 2024
	Petroleum hydrocarbons	Achieved 85% remediation depending on substrate and method	Dickson et al., 2020
*P. pulmonarius*	Total petroleum hydrocarbons (TPH), oil and grease	40–45% degradation at 6900–19000 mg/kg; effective on crude oil	Chiu et al., 2009; Olusola and Anslem, 2010; Njoku et al., 2017
*P. tuber-regium*	Crude oil	Efficient in degrading crude oil-contaminated soils; enhanced biodegradation and soil nutrient restoration	Isikhuemhen et al., 2003
	Total petroleum hydrocarbons (TPHs)	Reduced TPH (30.84%),	Adenipekun et al., 2011
*Polyporus* sp.	Crude oil/petroleum hydrocarbons	Effective degradation capacity.	Kristanti et al., 2011
*Trametes hirsuta*	Phenanthrene, Benzo[a]pyrene	91.26% and 87.72% degraded in 12 days	Hidayat and Yanto, 2018; Rathankumar et al., 2020
*T. versicolor*	PCP, BPA, phenols, chrysene, benzo[a]pyrene	>60% removal of several pollutants; enhanced degradation with plantain peel; BPA up to 90%	Wesenberg et al., 2003; Nguyen et al., 2014; Singh et al., 2019; Vipotnik et al., 2022

#### 3.1.2 Biodegradation of industrial dyes and persistent organic pollutants

Azo, anthraquinone, triphenylmethane, and heterocyclic polymeric dyes are synthetic dyes with large-scale uses in sectors such as textiles, cosmetics, pharmaceuticals, foodstuffs, and petroleum (Negi, 2025; Taha and Gouda, 2025). Azo dyes alone account for over half of all synthetic dyes produced annually. Despite their utility, the use of these dyes results in colored effluents that inhibit primary productivity, alter gas exchange in aquatic systems, and pose carcinogenic and toxic risks to human health (Alzain et al., 2023; Keshava et al., 2023; Saini and Choudhary, 2025). Industrial effluent contains non-biodegradable contaminating compounds like dyes and pesticides that persist in the environment and pose long-term environmental hazards. White-rot fungi such as *Phanerochaete chrysosporium* and *G. lucidum* produce oxidative and ligninolytic enzymes such as laccase, manganese peroxidase (MnP), and lignin peroxidase to break down recalcitrant pollutants such as azo dyes, PCBs, and other xenobiotics (Torres-Farradá et al., 2024; Boro et al., 2025).

*Phanerochaete chrysosporium* has a natural ability to biodegrade azo dyes into non-toxic metabolites (de Almeida et al., 2021; Tyagi et al., 2025), while *L. edodes* and *Ganoderma* spp. are effective in degrading endocrine-disrupting and pharmaceutical wastes (Muszyńska et al., 2018; Ghose and Mitra, 2022). Mushrooms are also utilized in mycoreactors for the decontamination of pesticides from textile wastewater and for large-scale wastewater treatment (El-Baz et al., 2024). Besides dyes, mycoremediation is a sustainable approach for pesticide degradation. Since the advent of DDT, pesticides such as insecticides, herbicides, and fungicides have been widely used in agriculture and public health (Pujiati et al., 2024; Pundir et al., 2024). These compounds persist in the environment and are toxic, causing widespread pollution that hinders soil fertility, disrupts microbial processes, and harms human health, with impacts including endocrine disruption, mutagenicity, and immunosuppression (Ali et al., 2021; Pathak et al., 2022; Garud et al., 2024).

Chlorinated insecticides such as aldrin, endosulfan, and DDT is particularly persistent. Ectomycorrhizal mushrooms, including *Gomphidius viscidus, Boletus edulis, L. bicolor*, and *Leccinum scabrum*, can biodegrade DDT (Álvarez et al., 2021; Mathur and Gehlot, 2021). *Agrocybe semiorbicularis, Flammulina velutipes, Hypholoma fasciculare, P. ostreatus*, and *Phanerochaete velutina* are among the mushrooms that biodegrade herbicides like diuron, atrazine, and terbuthylazine (Bhandari, 2017; Asad et al., 2025). *Pleurotus pulmonarius* biodegraded atrazine into less toxic metabolites, including deethylatrazine and deisopropylatrazine (Masaphy et al., 1993; Wyss et al., 2006). Collectively, these studies demonstrate the enzymatic potential of various mushroom species to transform industrially relevant dyes, POPs, and pesticides, forming the biochemical basis for mycoremediation. It is critical to distinguish this proven laboratory potential from the engineering challenge of creating reliable field-scale technologies. The data compiled in Table 4, while indicative of strong catalytic ability, are primarily derived from studies that simplify critical field constraints. They serve as a vital reference for the range of pollutants fungi can attack, but not as a direct predictor of cleanup timeframes or efficiency in complex environments.

**Table 4:** Bioremediation potential of mushroom species in degrading persistent organic pollutants (POPs) and synthetic dyes.

**Species**	**Pollutant degraded**	**Type (dye/POP)**	**Key findings**	**References**
*Agaricus bisporus*	Crystal violet, brilliant green	Dye	21.7 mg/g CV and 12.1 mg/g BG biosorbed at equilibrium	Pandey et al., 2013
	Levafix Braun E-RN, Acid Red 111, Basic Red 18		140.9 mg/g AR111, 400 mg/g BR18, 169.5 mg/g LB adsorbed	Toptas et al., 2014
*Coriolus versicolor*	Acid green, disperse red, basic orange		Up to 98% decolorization for AG	Sivasakthivelan, 2013
*Cordyceps militaris*	Reactive Yellow 18, Green 19, Red 31, Red 74		Up to 73.07% decolorization	Kaur et al., 2015
*Fomes fomentarius*	Methylene blue, rhodamine		232.73 mg/g MB and 25.12 mg/g Rhodamine adsorbed	Maurya et al., 2006
*Funalia trogii*	Synthetic dyes		Effective decolorization of textile dyes	Kapdan and Kargi, 2002
*Grifola frondosa*	Malachite green		Evaluated for MG degradation; % not specified	Rajput et al., 2011
*Irpex lacteus*	Chlorobenzaldehydes	POP	Efficient CB-CHO transformation via MnP	Šrédlová et al., 2021
*I. lacteus, Bjerkandera adusta, Pycnoporus cinnabarinus, Phanerochaete magnoliae*	PCBs		*B. adusta* showed the best soil-based degradation	Cvančarová et al., 2012; Chun et al., 2019
Jelly mushrooms	Malachite Green	Dye	Up to 99.75% degradation on Day 10	Rajput et al., 2011
*Lentinula edodes*	2,4-dichlorophenol	POP	Degradation enhanced with vanillin	Tsujiyama et al., 2013
	PCBs		Degradation potential confirmed	Ruiz-Aguilar et al., 2002; Chun et al., 2019
*Lentinus concinnus*	Red 60 dye	Dye	Adsorption: 92.6 mg/g	Bayramoglu et al., 2016
*L. tigrinus*	Chlorinated benzoates	POP	*In vitro* detoxification by microsomes	Stella et al., 2013
*L. strigellus*	Orange II, Crystal Violet	Dye	70% and 65% decolorization	Gomes et al., 2009
*Panus strigellus*	RB220, RBBR, MG		Up to 90% RB220 degradation	Cardoso et al., 2018
*Phanerochaete chrysosporium*	2,4-D, dyes	Dye, POP	Broad ligninolytic enzyme activity	Bumpus, 2004; Abatenh et al., 2017
	PCBs (Aroclor 1242, 1254, 1260)	POP	Effective in liquid, less in soil	Bumpus et al., 1985; Eaton, 1985; Chun et al., 2019
*Phlebia brevispora*	PCBs		Confirmed in the lab, limited soil testing	Kamei et al., 2006; Chun et al., 2019
*Phellinus igniarius*	Methylene blue, rhodamine	Dye	202.38 mg/g MB and 36.82 mg/g Rhodamine	Maurya et al., 2006
*Pleurotus eryngii*	Pesticides	POP	Degraded organophosphate and carbamate in soil	Abatenh et al., 2017
*P. fabellatus*	Direct blue 14	Dye	90.39% decolorization	Singh et al., 2013
*P. florida*	MG, coralene dyes, Blue CA, Black B133		Up to 99.5% decolorization	Sathiya et al., 2007; Rajput et al., 2011; Kunjadia et al., 2016
*P. ostreatus*	Pesticides, dyes, coralene yellow	Dye, POP	88% decolorization; enzymes active	Maurya et al., 2006; Kunjadia et al., 2016
	OH-PCBs, CB-OHs, CB-CHOs	POP	>80% OH-PCBs removed in 1 h	Šrédlová et al., 2021
	PCBs (Delor 103)		Up to 99.6% removal in 6 weeks	Cvančarová et al., 2012
	PCBs (soil trials)		~40% removal in 2 months	Zeddel et al., 1993; Kubátová et al., 2001; Chun et al., 2019
	Decabromo-diphenyl ethane		43.7% degraded in 30 days	Wang et al., 2022a
	DR5B, DB22, DB71, RB5	Dye	14.6–20.1 mg/g sorption	Alhujaily et al., 2020
*P. ostreatus, P. eryngii*	RBBR		86.1% and 77.9% adsorption, respectively	Lee and Tang, 2020
*P. pulmonarius*	MG, Victoria blue B, Brilliant blue		Up to 68.61% decolorization	Lallawmsanga et al., 2018
	RBBR, Congo Red, MB, Ethyl Violet		Laccase and MnP enhanced degradation	Bazanella et al., 2013
*P. sajor-kaju*	Malachite Green		Some decolorization on PDA	Rajput et al., 2011
*Pleurotus* spp.	Coralene dyes		88–98% degradation; multiple enzymes involved	Kunjadia et al., 2016
*Polyporous* sp. 1 and 2	Malachite Green		26.25–68.5% degradation	Rajput et al., 2011
*Pycnoporus sanguineus*	Azo dyes, phenolic pollutants	Dye, POP	Laccase-mediated breakdown	Harazono and Nakamura, 2005
*Rigidoporus lignosus*	Industrial dyes	Dye	Effective degradation and detoxification	Wesenberg et al., 2003
*Schizophyllum commune*	Malachite Green		Up to 97.5% degradation	Rajput et al., 2011
*Trametes hirsuta*	Blue CA, Black B133, Corazol Violet		56.98–91.1% decolorization	Sathiya et al., 2007
*T. versicolor*	Azo dyes, pesticides, and effluents	Dye, POP	Broad enzyme-based degradation	Wesenberg et al., 2003; Amaral et al., 2004; Abatenh et al., 2017
	PCBs	POP	Lab effective, with limited soil performance	Kubátová et al., 2001; Chun et al., 2019
	Pentachlorophenol (PCP)		>60% removal at 0.05 mg/kg	Nguyen et al., 2014

#### 3.1.3 Mycoremediation of agricultural pollutants

Pesticide and herbicide contamination in agricultural ecosystems is a significant risk to indicators for water quality and soil health. Fungi such as *Pleurotus, Ganoderma*, and *Lentinula* species can biodegrade organophosphates, carbamates, and atrazine into non-toxic metabolites (Maqbool et al., 2016). Unlike bacterial degradation, which often results in partial degradation or the formation of toxic intermediates, mycoremediation using mushrooms is a more efficient and cleaner detoxification process (Fu et al., 2025). *Pleurotus ostreatus*, for example, has been shown to degrade organochlorine pesticides and reduce their persistence in contaminated soils (Ahemad and Kibret, 2014). In addition, studies have demonstrated that *G. lucidum* can biosorb heavy metals such as lead and cadmium, aiding in the restoration of heavy metal polluted farmland (Gadd, 2004).

#### 3.1.4 Mycoremediation of industrial waste

White-rot and litter-decomposing fungi are potent mycoremediation agents through three general mechanisms: biodegradation, biosorption, and bioconversion. Their arsenal of ligninolytic and oxidative enzymes, such as lignin peroxidase (LiP), manganese peroxidase (MnP), laccase, cellulases, and xylanases, enables them to break down recalcitrant contaminants such as PAHs, nitrotoluenes, textile dyes, plastics, and pharmaceutical residues (Xie et al., 2017; Muszyńska et al., 2018). *Pleurotus* and *Trametes* species can degrade emerging pollutants, including oxytetracycline, bisphenol A, and lamotrigine, via extracellular enzymes, achieving mineralization yields of up to 90% under optimal conditions (Migliore et al., 2012; Chefetz et al., 2019). Notably, *P. pulmonarius* can immobilize nuclear waste when combined with Portland cement, demonstrating its remarkable versatility (Eskander et al., 2012). Biosorption utilizes dead or live fungal biomass to adsorb heavy metals (e.g., Cd, Pb) and xenobiotics through processes such as adsorption, ion exchange, and covalent binding. This process is facilitated by functional groups in cell wall compounds (chitin, glucans, proteins), and it is particularly suitable for industrial effluent treatment and the recovery of metals (e.g., Au, Cu) from wastewater. For instance, *Pleurotus* mycelium can remove 70% of lead ions within 24 hours (Ayele et al., 2021). The process loop is closed through bioconversion, which converts lignocellulosic wastes such as paper sludge and eucalyptus residues into value-added fungal biomass. Fungi such as *Pleurotus* and *Lentinula* thrive on challenging substrates, including cardboard waste and drug-contaminated media, producing edible or medicinal mushrooms (Brienzo et al., 2007; Kulshreshtha et al., 2014). This dual benefit, waste elimination and resource generation, aligns with circular economy principles. Challenges such as low degradation rates, competition with native microbiota, and issues of scalability remain, yet the field is promising. Current research focuses on solutions like genetically engineered strains (e.g., *Trametes* strains overexpressing laccase) and hybrid systems (e.g., fungi-bacteria consortia). For example, a consortium of *Phanerochaete chrysosporium* and *Pseudomonas* can improve PAH degradation by 40% (Sharma et al., 2022; Bokade and Bajaj, 2023). Table 5 provides an overview of the mycoremediation potential of selected mushroom species for various industrial and pharmaceutical wastes.

**Table 5:** Mycoremediation potential of selected mushroom species against various industrial and pharmaceutical wastes.

**Species**	**Waste category**	**Substrate/waste used**	**Key findings**	**References**
*Auricularia polytricha*	Industrial contaminants	Phthalates (BBP, DBP, DEP)	Enzymatic degradation via esterases/oxygenases	Chang et al., 2021
*Flammulina velutipes*	Textile agricultural waste	Ramie stalk textile waste	High yield (359 g) and biological efficiency (119.7%)	Xie et al., 2017
*Lentinula edodes*	Pharmaceutical waste	Piroxicam	Metabolizes piroxicam into less toxic aromatic byproducts	Muszyńska et al., 2018
		Azole fungicides	Partial degradation of azole fungicides showing moderate efficacy and biotransformation	Kryczyk-Poprawa et al., 2020
		Terbinafine	Transformed terbinafine into multiple identifiable byproducts	Kryczyk-Poprawa et al., 2020
*L. edodes, P. chrysosporium*		17α-ethinylestradiol	80% and 52% degradation by L. edodes and P. chrysosporium	Eldridge et al., 2017
*Pleurotus citrinopileatus, P. florida*	Industrial paper waste	Solid sludges, paper industry effluent	Capable of growing on and remediating cardboard and the handmade paper industry waste	Kulshreshtha et al., 2010, 2014
*P. ostreatus*	Pharmaceutical wastewater	Oxytetracycline (OTC)	Efficient degradation of OTC by extracellular enzymes with high mineralization efficiency	Migliore et al., 2012
		Dibutyl phthalate (DBP)	High DBP removal efficiency via enzymatic breakdown into low-toxicity compounds	Ahuactzin-Pérez et al., 2018
		Lamotrigine (LTG)	Transformed lamotrigine into an oxidized derivative, with potential for pharmaceutical wastewater treatment	Chefetz et al., 2019
		Vincristine and bleomycin	Removed by biosorption on fungal biomass	Jureczko and Przystaś, 2021
		Sulfonamide antibiotics	Removed up to 83–91% via biofiltration	Mayans et al., 2021
		Diclofenac, Bicalutamide, Lamotrigine, Metformin	Removed 43–73% from the water	Hultberg et al., 2020
	Organic pollutant	Chloro-hydroxyl-actones	Biotransformed to chlorolactones	Grabarczyk et al., 2020
	Personal care product	Triclosan	Fully degraded on day 1 via MnP and laccase	Mallak et al., 2020
	Fungicides	Carbendazim, thiophanate-methyl	Biodegraded fungicide residues	Sharma et al., 2020
	Industrial contaminant (plasticizer)	Benzyl butyl phthalate (BBP), DBP, DEP	Max degradation: DBP > BBP > DEP via esterases/oxygenases	Chang et al., 2021
*P. pulmonarius*	Radioactive/industrial waste	Radioactive cellulosic-based waste	Waste with mushroom mycelium solidified with Portland cement, forming a barrier to contaminant release	Eskander et al., 2012
*Trametes versicolor*	Chemical industrial waste	Bisphenol A (BPA)	Efficient enzymatic degradation of BPA, forming aromatic and aliphatic byproducts	Liu H. Y. et al., 2019
		4-tert-butylphenol	4-Tert-butylphenol detected at 14 mg/kg	Nguyen et al., 2014
	Soil organic matter components	Humic acids (HA)	Partial breakdown of humic acids into smaller humic-like substances, enhancing soil quality	Zahmatkesh et al., 2016
	Industrial plasticizer	Bisphenol A	Bisphenol A was detected at 26 mg/kg	Nguyen et al., 2014
	Industrial waste	4-Tert-octylphenol	4-Tert-octylphenol was removed by over 60%	Nguyen et al., 2014
	Industrial contaminant	Benzophenone	Benzophenone detected at 10 mg/kg	Nguyen et al., 2014
	Personal care product pollutant	Octocrylene	Octocrylene presence noted; concentration/removal not specified	Nguyen et al., 2014
*T. versicolor, Phanerochaete chrysosporium*	Polluted soil/industrial contaminants	Contaminated soils with lignin-like pollutants and xenobiotics	Degrade diverse organics using low-specificity ligninolytic enzymes; efficacy enhanced with carbon amendments	Rhodes, 2014

### 3.2 Heavy metal biosorption and bioaccumulation

Heavy metals are metallic elements with a density greater than 5 g/cm3 and the capacity to form sulfides. They primarily originate from anthropogenic sources, such as industrial processes, mining, iron and steel production, agriculture, transport, chemical industries, and household activities, leading to soil and environmental pollution (Ali and Khan, 2018; Laoye et al., 2025). Fungal bioremediation has been suggested as a green technology for the cleanup of heavy metal contamination, involving two main processes in mushrooms: biosorption, a passive and nonspecific metal binding to cell wall components, particularly chitin and melanin, and bioaccumulation, an energy-dependent active process mediated by membrane transporters, particularly Zrt/Irt-like proteins; this approach has been found to reduce metal bioavailability by 30–90% in contaminated environments (Yadav et al., 2021; Ab Rhaman et al., 2022). Mushrooms have enormous potential for the bioremediation of heavy metal-contaminated environments through biosorption and bioaccumulation mechanisms, thereby minimizing the bioavailability and environmental hazards of toxic metals, particularly lead (Pb), cadmium (Cd), mercury (Hg), and arsenic (Yadav et al., 2021; Ab Rhaman et al., 2022; Kondakindi et al., 2024).

Furthermore, mushrooms have evolved metal detoxification and survival mechanisms, including negatively charged cell walls that act as efficient cation exchangers for the adsorption of metals (Refaey et al., 2021; Verma and Srivastava, 2021). For example, *Pleurotus* species can remove metals, such as Pb, Cd, Ni, Hg, As, Fe, and Zn, from aquatic, terrestrial, and wastewater systems (Chen et al., 2022; Jamir et al., 2024). Saprotrophic fungi such as *Lentinus squarrosulus, Volvariella volvacea, Schizophyllum commune*, and *Auricularia auricula* have been reported to bioaccumulate multiple metals, including Hg, Fe, Zn, Pb, Cu, Ni, and Cd, demonstrating their significant potential for heavy metal bioremediation (Golian et al., 2021; Ab Rhaman et al., 2022; Liu et al., 2022).

#### 3.2.1 Mechanisms of heavy metal absorption in mushrooms

**Figure 4:**
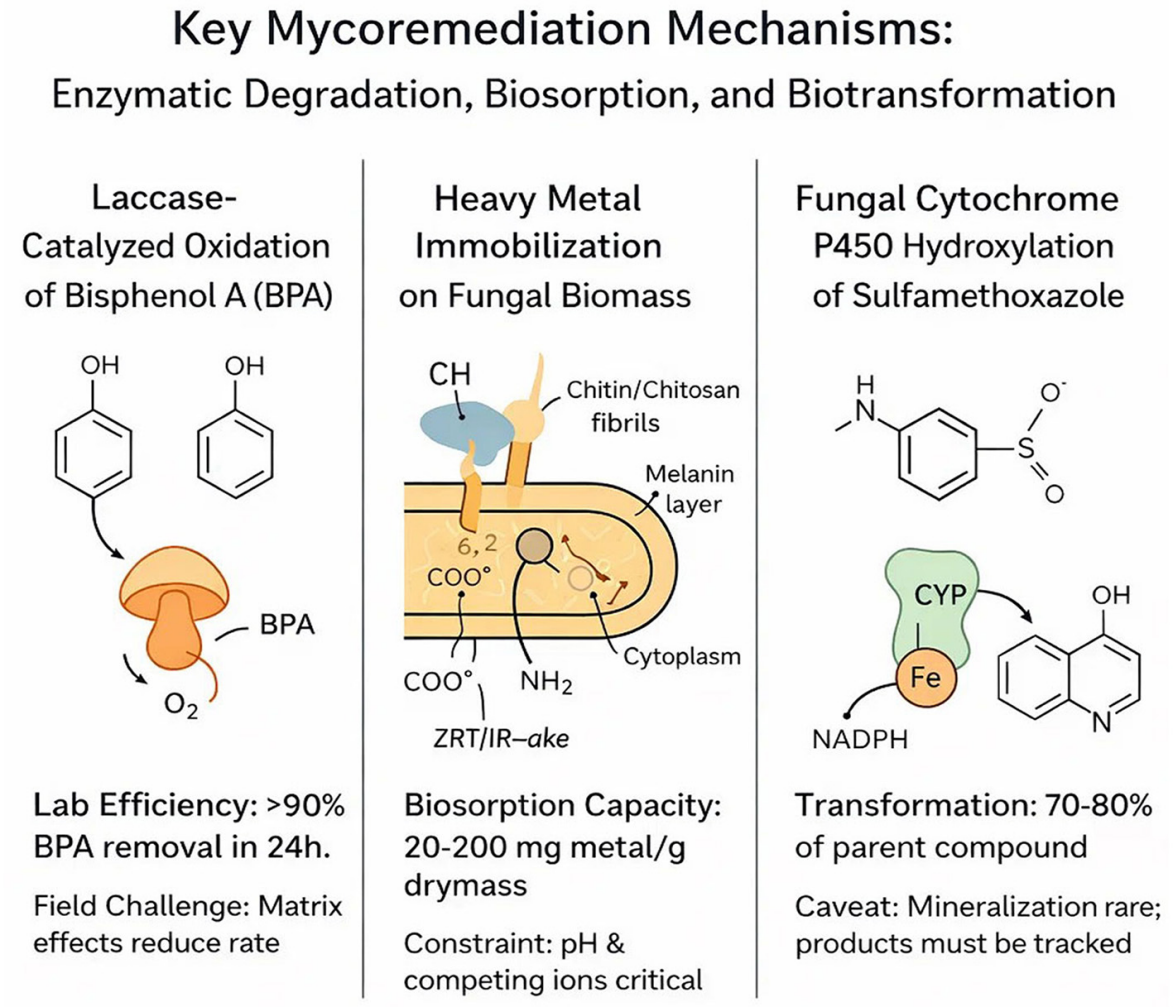
Mechanisms of fungal heavy metal absorption.

Mushrooms have different processes for the uptake, detoxification, and tolerance of heavy metals. These activities are broadly categorized into passive (biosorption) and active processes (bioaccumulation, enzymatic detoxification, and bioconversion), which are often controlled by species-specific traits and external factors (Figure 4).

**Biosorption: Physical and chemical passive metal binding**

Biosorption is a metabolism-independent process involving physical and chemical mechanisms such as passive ion exchange, adsorption, complexation, and precipitation, leading to the immobilization of heavy metal ions on cell wall components, particularly polysaccharides, proteins, and chitin (Kumar and Dwivedi, 2021; Zare et al., 2024). This process occurs on both dead and living mushroom biomass and is relatively fast. Notably, mushroom biomass upon death, being unconstrained by metabolic limitations, often exhibits higher metal-binding capacity (Kulshreshtha, 2018; Gül and Hung, 2025). In addition, extracellular precipitation by mobilized oxalates or phosphates can trap metals such as lead and arsenic (Gadd, 2010). Mushroom-based materials, including spent mushroom compost, dry fruiting bodies, and mycelial mats, have been used successfully as biosorbents (Kumar and Dwivedi, 2021; Periakaruppan et al., 2025).

This broad biosorptive capacity is attributed to functional groups such as hydroxyl, carboxyl, and amino groups, in the cell walls, which serve as active binding sites for metal ions (Dwivedi, 2023). Some species, such as *A. bisporus*, enhance metal retention by promoting the precipitation of calcium carbonate structures (Vimala and Das, 2009). Environmental factors (pH, temperature, metal ion concentration, and contact time) play a crucial role in biosorption efficiency (Shamim, 2018; Ameen et al., 2021). *Lentinula edodes*, for instance, has a high biosorption capacity due to its porous structure and ability to adsorb a wide range of impurities (Dong et al., 2019; Muñoz-Castiblanco et al., 2022). Furthermore, mushroom α-glucans and chitosan contribute to porosity and a high density of metal-binding sites for biosorption (Nowak et al., 2019).

**Bioaccumulation: Uptake and detoxification by facilitated transport**

An energy-dependent active process, bioaccumulation involves the accumulation of heavy metals by mushrooms through facilitated membrane transport, intracellular chelation with metallothioneins or glutathione, and vacuolar sequestration. This is achieved through the upregulation of metallothionein genes (e.g., *MT1, MT2*) in response to metal stress exposure and the action of vacuolar ABC transporters in metal compartmentalization (Sácký et al., 2022; Słyszyk et al., 2024). Hyperaccumulators like *Amanita rubescens* (Cd) and *P. ostreatus* (Pb) counteract high metal concentrations by the action of antioxidant enzymes (e.g., superoxide dismutase; Yadav et al., 2021). *Lentinula edodes* growth is inhibited by Cd at concentrations >50 mg/kg, while *P. ostreatus* can withstand Pb at ≤ 100 mg/kg (Yu et al., 2021). Certain mushroom-cultivating fungi like *L. edodes, P. ostreatus*, and *A. bisporus* can resist and tolerate high concentrations of metals under challenging environmental conditions, such as multi-metal exposure or freezing temperatures (Singh and Singh, 2024). They employ a combination of metal-binding proteins and antioxidant enzymes to counteract oxidative stress and maintain cellular integrity (Yadav et al., 2021; Dwivedi, 2023).

**Enzymatic detoxification and bioconversion**

Mushrooms detoxify harmful substances through enzymatic processes, utilizing oxidative enzymes such as peroxidases and laccases to transform heavy metals into less toxic or more stable forms. Although initially associated with lignin degradation and xenobiotic pollutant biodegradation, these enzymes are also involved in redox-catalyzed detoxification processes that enhance the fungus's tolerance to metal-induced stress (Kapahi and Sachdeva, 2017; Singh and Singh, 2024). These enzymatic systems also initiate bioconversion processes, enabling the biodegradation of lignocellulosic and industrial wastes alongside heavy metal sequestration. Their ligninolytic and cellulolytic enzyme systems enable them to degrade contaminated organic substrates, making them valuable agents in integrated waste valorization and bioremediation processes (Chen et al., 2022). The synergy between their ecological roles and metabolic versatility underscores the utility of mushrooms in green environmental detoxification. Field trials demonstrate metal uptake variations between wild and domesticated isolates (e.g., greater Cd in *Lactarius piperatus* than in cultivated) *A. bisporus* (Nagy et al., 2014). Mycorrhizal fungi (e.g., *Boletus edulis*) can modify the availability of metals in soil (Bolan et al., 2014). These traits are utilized in the mycoremediation of contaminated soil (Singh and Singh, 2024). Table 6 summarizes the enzymatic detoxification and bioconversion capacities of mushrooms, highlighting how their oxidative enzyme systems facilitate the transformation of toxic metals and organic wastes into less harmful forms.

**Table 6:** Heavy metal biosorption and bioaccumulation by mushrooms.

**Fungal material/ species**	**Target metal(s)**	**Maximum reported capacity or % removal**	**Primary mechanism**	**Optimal pH**	**Key findings and critical notes**	**References**
*Agaricus bisporus* (spent compost/biomass)	Cd^2^^+^, Pb^2^^+^, Zn^2^^+^	29.6 (Cd), 33.7 (Pb), 227% Zn increase in soil	Biosorption (chitin/chitosan)	5.5–6.5	Spent substrate boosts soil Zn uptake; pretreatment-dependent	Vimala and Das, 2009; Xu et al., 2012; Cheng et al., 2018; Sun et al., 2018b
	Ni^2^^+^ (soil)	Increased CH_4_ oxidation 3.8 ×	Biosorption/ Biostimulation	6–7 (soil)	Enhances microbial activity in contaminated soils	Sun et al., 2018a
	Cr^6+^	100% removal	Biosorption	2–3	Highly efficient in acidic media	Shukla and Vankar, 2015
	Multi-metal (Cd, Hg, Cu, Zn, Cr)	Effective bioaccumulator	Bioaccumulation	25–30 °C	Demonstrates broad uptake	García et al., 2005
*Pleurotus ostreatus* (mycelium/biomass)	Pb^2^^+^, U^6+^, Cu^2+^, Ni^2+^	53.7% (Pb), 84.5% (U), 68.5 mg/kg (Fe), 26.9 mg/kg (Cu)	Biosorption and bioaccumulation	5.0–6.0	High for U and Pb; field efficiency ~50–60%	Javaid et al., 2011; Tay et al., 2011; Zhao et al., 2016; Wang et al., 2019; Xu et al., 2021
	Cd^2^^+^, Hg^2^^+^, Co^2^^+^, Mn^2^^+^, Se, Fe, Zn	BAF > 1	Bioaccumulation and chelation	N/A	Tolerant; strong bioindicator	Milovanović et al., 2014; Muhammad et al., 2017a,b; Liu B. et al., 2019; Liu X. et al., 2019
	Co, Cu, Ni	Higher for Co	Biosorption	7.0, 28 °C	Selective biosorption	Mohamadhasani and Rahimi, 2022
*Lentinula edodes*	Cu^2^^+^, Cd^2^^+^	50.0 mg/g (Cu)	Biosorption (porous structure)	5.5–6.0	High surface area; Cd toxic >50 mg/kg	Chen et al., 2008; Hu et al., 2014; Yu et al., 2021
*Auricularia auricula* (modified biomass)	Cd^2^^+^, Pb^2^^+^, Cr^6+^	35.5 (Cd), 36.4 (Pb), 12.2 (Cr)	Enhanced biosorption	6.0 (Cd/Pb), 2–3 (Cr)	Chemical modification boosts capacity	Dong et al., 2017; Song et al., 2017a,b; Jin et al., 2020
*Ganoderma lucidum*	Pb, Ni, Zn, Cd, Cu	138, 3.48, 29.8, 1.01, 3069 mg/kg	Bioaccumulation and chelation	5–6	Accumulates metals strongly	Ipeaiyeda et al., 2020
	Cu, Pb, Co	Broad accumulation	Both	5–6, 28 °C	Multi-metal potential	Das, 2005
*Flammulina velutipes* (spent compost)	Cu^2^^+^, Zn^2^^+^, Hg^2^^+^	Effective biosorption	Biosorption	5–7	Low-cost biosorbent	Luo et al., 2013; Qu et al., 2015; Kumar and Dwivedi, 2021

#### 3.2.2 Mycorrhizal mushrooms and heavy metal immobilization

Mycorrhizal fungi contribute to heavy metal remediation through symbiotic associations with plant roots, thereby immobilizing metals in soil. ECM fungi such as *Suillus luteus, Amanita muscaria*, and *Hebeloma crustuliniforme* can accumulate toxic metals within their mycelium, thereby reducing metal uptake by plants and decreasing their mobility in the contaminated environment (Colpaert et al., 2011). The extensive hyphal networks of these mushrooms act as biological filters, immobilizing and retaining heavy metals while also promoting plant growth in polluted soils (Krznaric et al., 2009).

Laboratory studies have demonstrated that *S. luteus* can suppress lead (Pb) transfer to wheat by 40% in contaminated soil. However, this efficiency is reduced under acidic conditions (pH < 4.5), which can mobilize the metal (Krznaric et al., 2009). Furthermore, AMF, such as *Glomus intraradices*, enhance phytoremediation by improving plant tolerance to metal toxicity, increasing metal uptake, and facilitating immobilization in fungal structures (Garg and Bhandari, 2016). These symbiotic interactions represent a sustainable, natural strategy for the rehabilitation of industrially and agriculturally polluted soils.

#### 3.2.3 Saprotrophic mushrooms and heavy metal bioaccumulation

Certain saprotrophic mushrooms possess high biocapacity for heavy metal accumulation and, therefore, are valuable assets in mycoremediation. *Pleurotus ostreatus* demonstrates 80% removal of Hg in laboratory experiments, but under field conditions, this is lowered to 50–60% as a result of interference caused by organic matter (Jamir et al., 2024). Spent mushroom compost reduces the cost of bioremediation by 30% in industrial implementation (Kumar and Dwivedi, 2021). These mushrooms achieve bioaccumulation through cell wall-binding mechanisms, where chitin, glucans, and melanin act as metal-binding molecules, sequestering and detoxifying harmful elements (Wang et al., 2019). Experiments have demonstrated that *L. edodes* has the ability to remove large amounts of cadmium and lead from industrial wastewater, rendering it perfect for mass-scale bioremediation purposes (Yu et al., 2021). Similar is the case of *P. ostreatus*, which has been shown to bioaccumulate and be tolerant to mercury and offer a natural method of alleviating mercury contamination in aquatic and terrestrial ecosystems (Gadd, 2004). The high biosorption capacities reported in laboratory studies (Table 7) are a measure of a fungus's intrinsic physicochemical ability to bind metal ions, often using purified biomass in simple aqueous solutions. This should not be equated with predictable remediation performance in soils or wastewater, where factors like organic matter, pH fluctuations, and the presence of competing ions can drastically reduce uptake efficiency and longevity. Table 7 provides a comparative overview of this fundamental biosorption potential across mushroom species, the metals targeted, and the optimized laboratory conditions under which these capacities were determined.

**Table 7:** Integrated fungal bioprocesses for a circular bioeconomy, demonstrating the synergistic conversion of waste streams into value-added products (food, feed, bioenergy, biomaterials) and soil amendments for enhanced carbon sequestration.

**Application pathway**	**Fungal agent/process**	**Input (waste stream)**	**Primary output(s)**	**Key performance metric/finding**	**References**
Waste valorization and food production	Cultivation of *Pleurotus* spp. (e.g., *P. ostreatus, P. florida*)	Agro-industrial residues: wheat straw, rice straw, banana leaves, cotton stalks	Edible mushroom biomass	Biological Efficiency (BE): 80–120% on various substrates; converts low-value waste to nutritious food	Philippoussis et al., 2001; Jonathan et al., 2008; Adedokun and Akuma, 2013; Prasad et al., 2018; Sardar et al., 2020; Akter et al., 2022
	Cultivation of *Lentinula edodes* (Shiitake)	Hardwood sawdust, supplemented substrates	Edible mushroom biomass	Successful cultivation on lignocellulosic waste produces a high-value product	Rani et al., 2008; Lin et al., 2015
	Cultivation of *Volvariella volvacea* (Paddy straw mushroom)	Paddy straw, banana leaves, oil palm empty fruit bunches	Edible mushroom biomass	Adapted to tropical conditions; efficient waste converter	Belewu and Belewu, 2005; Triyono et al., 2019
	Cultivation of *Auricularia* spp.	Cotton stalks, various crop residues	Edible mushroom biomass	Grows on diverse agricultural wastes	Vega et al., 2022
Bioenergy production	Fungal pretreatment for bioethanol: *Pleurotus* spp., *Trametes* spp.	Lignocellulosic biomass: wheat straw, maize straw, rice husk, sawdust	Pretreated biomass for enzymatic hydrolysis and fermentation → Bioethanol	Ethanol yield: 150–300 L/ton biomass after fungal delignification	Bilal et al., 2017; El-Imam et al., 2021; Fasiku and Wakil, 2021; Saini et al., 2022; Shankar et al., 2024; Yin et al., 2025
	Biogas from spent substrate: anaerobic digestion (AD) of SMS	Spent mushroom substrate (SMS) from *Pleurotus, Agaricus*, etc.	Methane-rich biogas	Methane yield: 200–300 L/kg volatile solids from SMS; integrates waste management with energy production	Mehta et al., 1990; Mustafa et al., 2016; Lalak et al., 2016; Sethumadhavan and Selvan, 2018; Kumar et al., 2021; Kumar P. et al., 2022; Kumar V. V. et al., 2022; Wang et al., 2022b,c
	Integrated solid-state digestion: mushroom cultivation coupled with AD	Palm kernel cake, empty fruit bunches, other tough biomass	Mushrooms + enhanced biogas yield from pre-digested residue	Increases overall energy recovery from waste streams	Mamimin et al., 2021; Panngoeen et al., 2023
Mycelium-based biomaterials	Mycelium growth and binding (*Ganoderma, Trametes, Pleurotus, Schizophyllum*)	Agricultural residues (straw, husks, sawdust) bound by mycelium	Biodegradable composites for packaging, insulation, textiles, and construction	Compressive strength: 0.1–0.3 MPa; thermal conductivity: ~0.07 W/m·K; fire-resistant; degrades in weeks/months	Siddique et al., 2016; Attias et al., 2017, 2020; Xing et al., 2018; Girometta et al., 2019; Elsacker et al., 2020; Appels and Wösten, 2021; Sivaprasad et al., 2021; Rajendran, 2022
Soil amendment and carbon sequestration	SMS application as organic fertilizer/amendment	Spent Mushroom Substrate (SMS)	Improved soil structure, fertility, microbial biomass, and soil organic carbon (SOC)	Can sequester 0.5–2.0 t CO_2_-eq ha^−1^ year^−1^; increases water retention and crop yield	García-Delgado et al., 2015; Meepun and Siriket, 2019; Liu et al., 2021; Zhang W. et al., 2023; Chen L. et al., 2024; Tang et al., 2024
	Mycorrhizal inoculation: ECM (*Pisolithus, Laccaria*)/AMF (*Rhizophagus*) in agroforestry	Native or degraded soils	Enhanced plant growth, drought resilience, nutrient uptake, and soil carbon stabilization	Increases plant survival and productivity in stressed environments; promotes long-term carbon storage	Bender et al., 2016; Fall et al., 2022; Chen B. D. et al., 2024
Biocontrol and phytoremediation support	Use of fungal extracts or spent substrate	SMS or fungal cultures	Biopesticidal compounds; enhanced plant health and phytoremediation of metals	Reduces pathogen incidence and insect pests; improves heavy metal uptake by plants when combined	-

### 3.3 Critical evaluation of methodologies in mycoremediation research

The impressive degradation percentages reported in Tables 4–7 position fungi as powerful remediation agents. However, the translational gap between laboratory promises and field application is vast, largely due to methodological oversimplifications in standard research protocols.

#### 3.3.1 The “optimized conditions” fallacy

High degradation rates for pollutants like PAHs (e.g., >90% for *Pleurotus ostreatus*) are almost universally achieved under optimized, small-scale conditions: sterile or autoclaved soil/water, single contaminants, ideal moisture, temperature, and pH, and with ample nutrient amendments (Rathankumar et al., 2020; Zhuo and Fan, 2021). These conditions negate the key challenges of field remediation: microbial competition, predation, heterogeneous contaminant mixtures, low nutrient availability, and suboptimal environmental conditions. Studies that transition to non-sterile, historically contaminated matrices consistently report lower and more variable efficiencies (Cvančarová et al., 2012; Mayans et al., 2024).

#### 3.3.2 Incomplete analysis of pollutant fate

A major methodological shortcoming is the reliance on “removal” metrics based on parent compound disappearance (e.g., via GC-MS). This ignores the formation and potential toxicity of transformation products, such as PAH-quinones or chlorinated intermediates from PCB degradation. Without employing non-target high-resolution mass spectrometry or toxicity assays (e.g., Microtox^®^) throughout the experiment, studies risk reporting a successful degradation that merely converts a parent pollutant into a different, yet still harmful, suite of compounds (Šrédlová et al., 2021).

#### 3.3.3 Enzymatic activity vs. *in situ* function

The attribution of degradation to specific enzymes (laccase, MnP, LiP) is often based on *in vitro* assays of culture supernatants under ideal pH. There is a paucity of data directly linking the *in-situ* expression and activity of these enzymes within a complex, contaminated soil matrix to the observed pollutant removal. Furthermore, many studies use high, often unrealistic, contaminant concentrations that can be toxic even to fungi, skewing the results away from realistic remediation scenarios.

#### 3.3.4 Lack of standardized consortium testing

Most research focuses on single fungal species. In natural environments, degradation is carried out by consortia. The methodological approach to designing and testing stable fungal-bacterial or fungal-fungal consortia is not standardized. Simple co-culture experiments fail to replicate the spatial structure and nutrient gradients of soil, limiting their predictive value for field application.

### 3.4 Role of mycorrhizae in remediation of soil

Mycorrhizal fungi occupy the center of ecological processes in soil fertility, plant nutrient acquisition, and ecosystem resistance. Mycorrhizal fungi support ecologically sound agriculture, conservation, and environmental restoration by enhancing better soil anchorage, drought alleviation effects (Zhang et al., 2018; Kalamulla et al., 2022), and mitigation of adverse impacts of intensive production systems (Sarwade et al., 2024; Andres et al., 2025). Mycorrhizae develop symbiotic associations with plant root systems and trigger phosphorus, nitrogen, and other nutrient uptake in exchange for carbon end-products of plant byproducts. Mycorrhizae break down organic contaminants, immobilize heavy metal ions in roots, and create resistance in plants to toxicity at the molecular level (Suárez et al., 2023; Wahab et al., 2023; Rigobelo, 2024).

Various species including AM, and ericoid (ERM) fungi, exhibit metal deposition in extramatrical hyphae, thus reducing translocation to the aerial part and achieving heavy metal tolerance. ECM fungi are also employed as soil pollution bioindicators (Chot and Reddy, 2022; Polussa et al., 2024; Shi et al., 2024). Mycorrhizae thrive under stressful situations, i.e., post-fire in the forest, by enhancing uptake and growth (Policelli et al., 2020; Elakiya et al., 2023; Dyshko et al., 2024). They take up organic and inorganic pollutants, enhance soil fertility, and include agriculture (Ahmed et al., 2025; Chaudhary et al., 2025). But their intrinsic mechanisms have been diverted because of overuse of agrochemicals (Pagano et al., 2023; Chaudhary et al., 2025). Precise use of artificial mycorrhizal inocula and host-fungus relationships is thus advised in a bid to maintain soil fertility at least with external supplies.

### 3.5 Synthesis and knowledge gaps in mycoremediation

While the enzymatic toolbox of white-rot fungi is remarkably effective against diverse pollutants in controlled settings, field-scale application faces a “black box” challenge. The interactions between introduced fungal inoculants, indigenous microbial communities, soil physicochemical properties, and plant roots ultimately determine remediation success. Key gaps include understanding how to engineer stable, competitive fungal-bacterial consortia, how to maintain fungal enzymatic activity in non-sterile, heterogeneous environments, and how to accurately assess the formation and ecological risk of transformation products during partial pollutant degradation. Closing these gaps is essential for transitioning mycoremediation from a promising laboratory technology to a reliable, *in-situ* cleanup strategy.

## 4 Integrated applications: from core mechanisms to circular solutions

**Figure 5:**
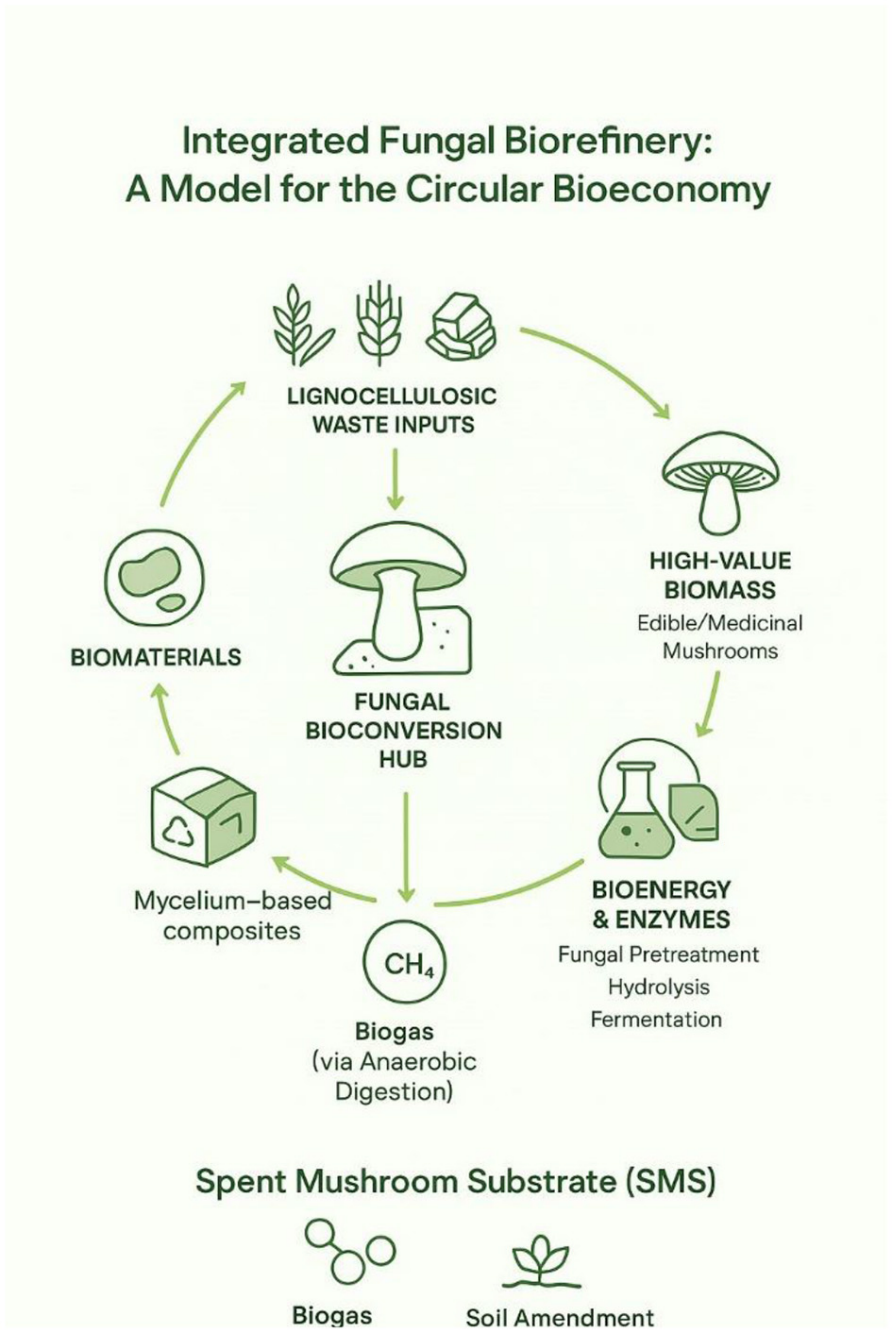
Integrated fungal biorefinery: a model for the circular bioeconomy.

Building on the principle of using fungi for environmental cleanup, their remarkable enzymatic arsenal can also be directed toward the valorization of waste streams, creating a circular economy that directly displaces fossil fuels. This section explores the cultivation of mushrooms on industrial and agricultural waste and the subsequent conversion of both fungal biomass and spent substrate into next-generation biofuels. The integration of these applications into a cascading circular system is conceptualized in Figure 5, which illustrates the fungal biorefinery model.

Table 7 presents integrated fungal bioprocesses within a circular bioeconomy framework, illustrating how diverse fungal systems convert agricultural and industrial waste streams into value-added products, including food, feed, bioenergy, biomaterials, and soil amendments that enhance carbon sequestration.

### 4.1 Waste valorization for bioenergy and feed

Industrial and agricultural lignocellulosic wastes are widely proposed as inexpensive substrates for mushroom cultivation, aligning with circular economy principles. However, the consistent and efficient bioconversion of these heterogeneous, often variable wastes into high yields of mushroom biomass remains a significant bioprocessing challenge. Factors such as substrate pre-treatment requirements, contaminant suppression, and the need for strain-specific substrate formulations can offset the theoretical cost advantage and hinder reliable scale-up. During bioconversion, the mushrooms degrade these waste products catabolically by depolymerizing cellulose, hemicellulose, and lignin, which are complex polymers, into simpler forms. White-rot fungi like *Pleurotus* spp. and *L. edodes* are strongly effective in bioconverting agro-industrial wastes (straw, sawdust, fruit peels) into edible biomass. Their complement of enzymes like laccases, peroxidases, and cellulases, breaks down lignocellulose to biological efficiencies (BE) of 123% on wheat straw (Philippoussis et al., 2001). Spent substrate (SMS) also supports sustainability as a soil amendment, adding fertility and sequestering 0.5–2.0 t CO_2_/ha/year (Zhang Y. et al., 2023). Evidence of integration in circular bioeconomies exists with pilot plants utilizing 1 t/day of waste to produce 300 kg of mushrooms (Dinakarkumar et al., 2024), even though substrate pretreatment remains one of the biggest challenges for lignin-rich wastes (Gałazka et al., 2025; Ibarra-Rondón et al., 2025).

Aside from recycling wastes, mushroom farming on industrial wastes also promotes sustainable agriculture because it reduces the application of synthetic fertilizers and landfill materials. Furthermore, SMS, or the spent biomass after harvesting, can be utilized as soil amendment to improve the fertility and structure of the soil (Kumar P. et al., 2022; Beig and Shah, 2023). Particularly, mitigation of climate change by reducing the greenhouse gas emission of decomposing organic wastes in landfills and enhanced carbon sequestration via use of spent substrates as a soil amendment (Zhang Y. et al., 2023) are obtained using this bioconversion process. The integration of mushroom bioconversion processes within circular bioeconomy frameworks has high potential for waste valorization, resource efficiency, and sustainable agri-food production (Kulshreshtha et al., 2014; Dinakarkumar et al., 2024).

### 4.2 Mycelium-based biodegradable materials

**Figure 6:**
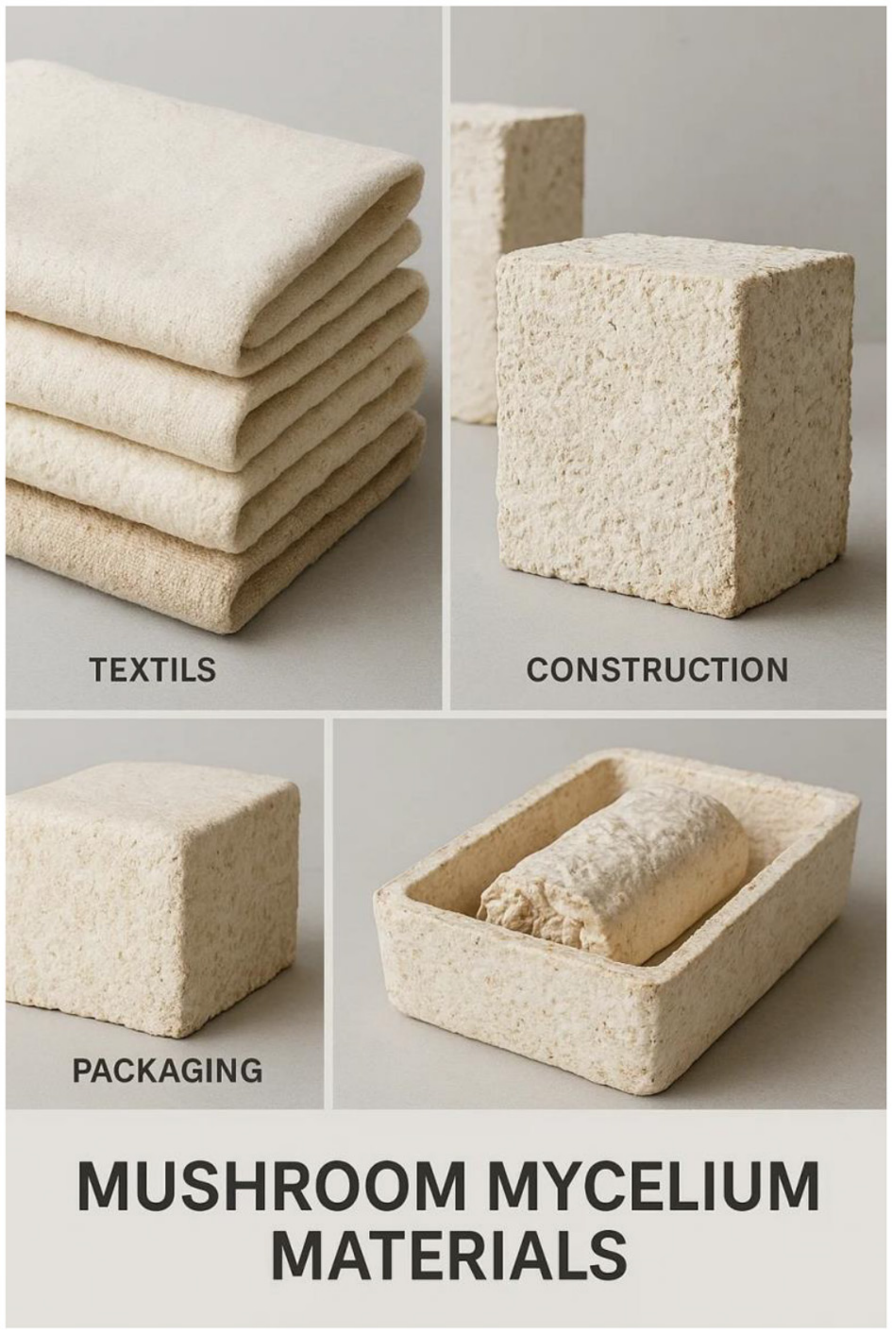
Applications of mushroom mycelium-based materials.

Mycelium-based materials represent a promising class of biofabricated alternatives to conventional plastics and synthetic foams, leveraging the ability of fungal mycelium to bind lignocellulosic waste into cohesive composites (Figure 6). Despite their compelling sustainability narrative, widespread commercialization is constrained by limitations in material properties such as susceptibility to water degradation, variable mechanical strength, and inconsistent production quality that must be solved to meet industry specifications for durability and performance. Solid-state fermentation induces fungi like *Fomes fomentarius, G. lucidum*, and *P. ostreatus* to grow compact, entangled matrices of hyphae that encapsulate the substrate into cohesive moldable composites (Rajendran, 2022; Angelova et al., 2025).

A key benefit of mycelium products is their biodegradability; they break down completely in soil within weeks or months, unlike petrochemical plastics, which persist for centuries. Mycelium materials also self-renew, are light in weight, are fire-resistant, and have variable mechanical characteristics namely compressive strength, flexibility, and thermal insulation depending on the fungus type and substrate composition (Attias et al., 2020; Sivaprasad et al., 2021).

However, it is important to acknowledge the current limitations of mycelium materials. Issues of vulnerability to moisture and water damage, durability relative to synthetics over long time-scales, and inhomogeneity across production batches are the subject of ongoing research and development. Despite these challenges, mycelium composites are already seeing commercial success in several limited markets. For example, they can be used to replace Styrofoam for packages and cushions, with the potential to save up to 90% in embodied energy (Zoungrana et al., 2025).

For the textile industry, mushroom-based leather substitutes such as *T. versicolor* and *P. ostreatus* are elastic, biodegradable, and non-violent toward animals and possess tensile strengths ranging from 15–40 MPa (d'Errico et al., 2024). For building construction, bricks and boards from species like *S. commune* and *G. sessile* possess a thermal conductivity of 0.07 W/mK or less and can be employed for energy conservation in building insulation (Attias et al., 2020). Secondly, mycelium production realizes circular economy goals by converting agro-industrial by-products into low-carbon added-value products. In an era where the globe is rapidly concentrating on microplastic pollution, fossil fuel consumption, and global warming, industrial scale bioproduction of mycelium composites are a cleaner technology future that aligns biotechnology, material science, and environmental design in substituting non-biodegradable pollutants with living and self-healing alternatives.

### 4.3 Fungal amendments for sustainable agriculture

Fungi play an important role in the development of sustainable agriculture by enhancing soil fertility, enhancing crop quality, and reducing the application of chemical pesticides and chemicals. Saprotrophic fungi, for instance, *P. ostreatus, A. bisporus*, and *L. edodes* enhance soil fertility and nutrient flow by breaking down SMS into nitrogen-, phosphorus-, and microbe-rich organic soil fertilizer. They improve root growth, microbial diversity, and crop yields, while minimizing environmental degradation (Liu et al., 2021; Tang et al., 2024). Mushrooms like *L. bicolor* and *Pisolithus tinctorius* root association improves phosphorus and nitrogen absorption and plant and soil tolerance of stress and drought. Their hyphae symbioses form soil accretion and carbon sequestration and therefore play a critical function in agroforest- and forest-maintenance systems (Chen B. D. et al., 2024).

Aside from that, *P. pulmonarius* and *G. lucidum* are also in the spotlight regarding whether they can potentially find their place in biopesticide manufacture. They are the producers of insecticidal and antifungal bioactive compounds, particularly, ganoderic acids and pleurotin and present eco-friendly substitutes for chemical pesticides (Girometta et al., 2019; Nguyen et al., 2022). Mushroom technology interface and regenerative agriculture drive climate-resilient agriculture with the green process that harmonizes food security and ecosystem health.

## 5 Synthesis and knowledge gaps in mycelium materials

Mycelium composites represent a transformative development in material science, but their path to replacing conventional plastics and foams is constrained by property limitations (e.g., hydrophobicity, long-term durability) and production variability. The central scientific challenge is to control the structure-property relationship precisely. This requires foundational research linking fungal genetics and morphology (e.g., hyphal branching, density) to the macroscopic mechanical and functional properties of the final composite. Additionally, developing standardized, scalable cultivation and post-processing techniques that ensure consistent quality is a critical engineering gap that must be addressed to meet industrial specifications.

### 5.1 Bioenergy production

**Figure 7:**
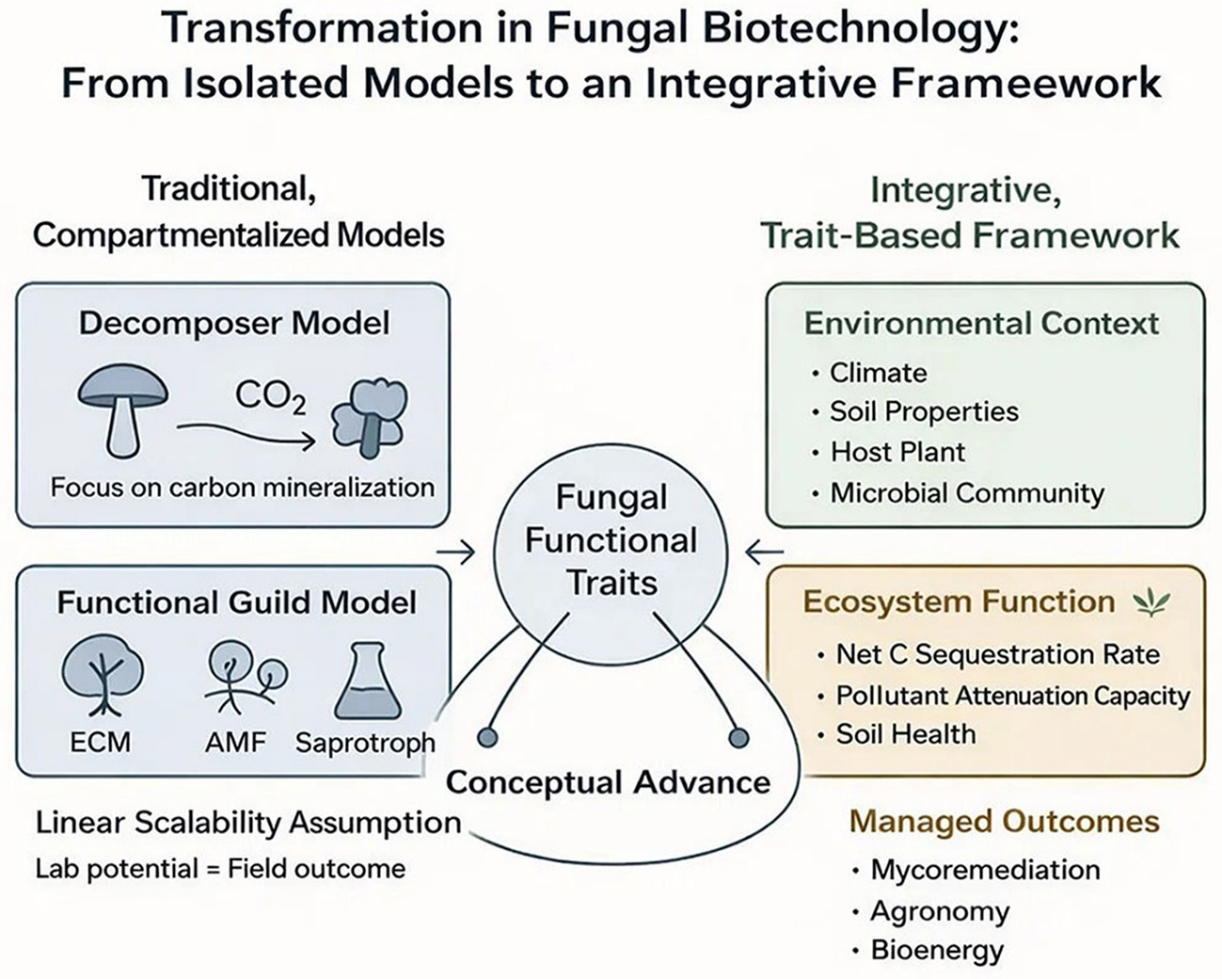
Conceptual evolution in understanding fungal roles in the carbon cycle and environmental remediation.

Fungal biotechnology is integral to the lignocellulosic bioethanol pipeline, primarily by providing lignin-modifying enzymes that pretreat recalcitrant plant biomass (Ahmed et al., 2020; Zhu et al., 2024). The central challenge for this fungal pretreatment, however, is economic: the process is often slower and less amenable to continuous industrial operation than established physicochemical methods (e.g., steam explosion, acid hydrolysis), raising concerns about capital costs and volumetric productivity at scale (Ahmed et al., 2020; Zhu et al., 2024). The present production technology uses diversified agricultural and forest residues like sugar-containing materials like sugarcane molasses, starch crops like wheat and corn, and lignocellulosic biomass of crop residue and wood waste (Stefi and Vorgias, 2025). Among the feedstocks mentioned, lignocellulosic biomass is full of promise since it is renewable and abundant. Apart from bioethanol, mushrooms are further employed in anaerobic digestion of spent substrate for biogas production and pyrolysis for bio-oil production, hence utilized in massive amounts in the bioenergy industry (Leong et al., 2022). Mushrooms, in this case, white-rot fungi such as *Ganoderma, Pleurotus*, and *Trametes*, play an integral role in the conversion of biofuels by releasing ligninolytic enzymes (e.g., laccase, lignin peroxidase, and manganese peroxidase) which efficiently depolymerize recalcitrant plant polymers to fermentable sugars (El-Ramady et al., 2022). Microbial fermentation is used to convert the sugars into bioethanol to obtain a green fungal-mediated bioconversion process (Yoon and Lee, 2022). Figure 7 illustrates the production of biofuel by various mushroom species and their application in synthesizing bioethanol and biogas by various substrates and processes.

Lignocellulosic biomass pretreatment remains the most critical step toward bioethanol production with severe cost and efficiency implications accounting for around 30–35% of the cost (Yin et al., 2025). Fungal pretreatment is less toxic compared to the traditional method with lower toxic chemical usage and its potential to yield more sugar. Through the application of agricultural waste namely wheat straw, corn stalk, and sawdust, mushroom-mediated bioconversion facilitates a circular bioeconomy that not only avoids greenhouse gas emissions but also enhances the conversion of low-value organic wastes to clean energy (Althuri and Mohan, 2022). Through this, it supports global climate change mitigation through the combination of waste valorization with clean energy generation. Further studies in fungal biotechnology and fermentation technology must be pursued to ensure yield maximization and scale-up. *Pleurotus* spp., for instance, have been found to possess exceptional ability to adapt to submerged and solid-state fermentation systems and are therefore model organisms for industrial large-scale bioethanol production (Xu et al., 2022). Yet, further research must be carried out for enhanced encouragement of pretreatment technologies and microbial strains, followed by cost-effectiveness and sustainability. Through enabling the symbiosis of fermentative microbes and fungal enzymes, this integrated strategy is not only able to make a big impact on a low-carbon energy era but also alleviate concerns about farm waste management (Ramamoorthy et al., 2023).

Despite the high yields, scalability of the method is hampered by several techno-economic and sustainability factors. The critical techno-economic analysis (TEA) indicates that fungal pretreatment is inferior regarding time expenditure relative to the conventional chemical (acid/alkali) and physicothermal (steam explosion, AFEX) strategies. This can result in larger reactor volumes and increased capital costs in a continuous industrial process. Furthermore, the life cycle evaluation of the entire value chain (i.e., from substrate preparation and fungal cultivation to enzyme production, hydrolysis and fermentation) needs to be considered. Key factors include the energy balance of the production process, the environmental consequences of discarding spent mushroom substrate (SMS) and the greenhouse gas (GHG) emissions in comparison to fossil fuels and other biofuel methods. Research should focus on simultaneously improving the economic and environmental performance of fungal biorefinery processes.

### 5.2 Synthesis and knowledge gaps in fungal biorefining

The integration of mushroom cultivation into waste valorization and biofuel production embodies circular economy principles. However, its economic feasibility is hampered by the time and space intensity of fungal solid-state fermentation compared to chemical/thermal pretreatment. Research must focus on process intensification, such as developing faster-growing or more enzymatically aggressive fungal strains, optimizing reactor designs for solid-state processes, and creating cascading systems in which the fungal pretreatment stage itself produces a valuable product (e.g., mushrooms or enzymes). Furthermore, comprehensive techno-economic analysis and life-cycle assessment (LCA) are needed to objectively compare fungal-based biorefineries with conventional alternatives across environmental and economic metrics.

## 6 Reconceptualizing fungal roles and future pathways in climate mitigation

The evidence compiled in this review does more than catalog fungal functions; it challenges and refines foundational concepts in fungal ecology and biogeochemistry. Moving beyond a compartmentalized view of fungi as decomposers, mutualists, or bioremediation tools, a synthesis of their roles reveals how they act as integrated engineers of the carbon cycle. This section first articulates how current knowledge advances our conceptual understanding of fungal functions (6.1), and then outlines the convergent research framework required to translate this new understanding into climate solutions (6.2). We propose a convergent research framework (Figure 7) built on four synergistic pathways to unlock the systemic potential of fungal climate solutions.

### 6.1 Reconceptualizing core fungal functions in the carbon cycle

#### 6.1.1 From decomposers to carbon fate engineers

Traditional fungal ecology has largely been framed by the decomposer-dichotomy model, which emphasizes the catabolic role of fungi in breaking down organic matter and releasing CO_2_. The data synthesized here necessitate a broader, more consequential view: fungi are primary engineers of carbon fate. Their defining influence lies not merely in whether carbon is decomposed, but in the biochemical pathway it follows. The same ligninolytic enzymes that mineralize plant litter also drive the polymerization of stable humic substances. Melanized hyphae from mycorrhizal and saprotrophic fungi contribute disproportionately to persistent MAOC. Furthermore, the “Gadgil effect”—whereby mycorrhizal fungi suppress free-living decomposers, illustrates that fungi can regulate carbon turnover not only directly but indirectly through microbial competition. This reframing positions fungi at the central decision point of the terrestrial carbon cycle, determining the allocation of plant-derived carbon between rapid atmospheric recycling and long-term soil sequestration.

#### 6.1.2 Beyond functional guilds: toward a trait-based continuum

A second conceptual advance necessitated by this review is the move beyond rigid functional guild classifications (e.g., ectomycorrhizal vs. saprotrophic) as predictors of ecosystem outcome. While useful heuristics, these categories obscure significant functional overlap and intra-guild diversity. For instance, some ECM fungi (e.g., *Paxillus involutus*) retain oxidative capabilities to access organic nitrogen, directly decomposing soil organic matter, while some saprotrophs (e.g., *Ganoderma* spp.) are efficient humifiers that stabilize carbon. A more predictive framework focuses on key functional traits: enzymatic portfolios (e.g., laccase vs. peroxidase expression), hyphal properties (melanization, hydrophobicity), and metabolic trade-offs (growth rate vs. carbon use efficiency). This trait-based lens explains why a melanized saprotroph in a cold climate may contribute more to persistent carbon pools than a non-melanized ECM fungus in a nitrogen-rich system. Embracing this continuum is crucial for accurately modeling fungal community effects on carbon cycling and for selecting the right fungal phenotypes, regardless of guild, for targeted climate solutions.

#### 6.1.3 From laboratory potential to ecosystem emergence

A dominant, often implicit, framework in applied mycology is the linear scalability assumption: that a function quantified in a controlled, optimized laboratory setting (e.g., 95% pollutant degradation, high biosorption capacity) will translate predictably to field efficacy. This review systematically demonstrates why this assumption is flawed. Fungal community function is an emergent property of complex interactions, fungal-bacterial competition, predation by microfauna, feedback with plant hosts, and response to physicochemical heterogeneity, that are absent in reductionist lab studies. The high degradation percentages in Table 4 or the precise carbon allocation figures in Section 2.1 are measures of physiological potential, not forecasts of *in-situ* performance. Therefore, the central challenge for fungal biotechnology is not merely discovering potent strains, but engineering the ecological context, through consortium design, substrate conditioning, or habitat management to enable the expression of that potential in the field. This represents a fundamental shift from a microbiology-centric to an ecology-centric application framework. These conceptual advances provide the necessary foundation for the next phase: developing tools and strategies to harness these fungal functions deliberately. This leads to the following integrative research model.

### 6.2 An integrative research framework for fungal climate solutions

The future of fungal biotechnology lies in consciously merging ecological principles with cutting-edge technology.

**Figure 8:**
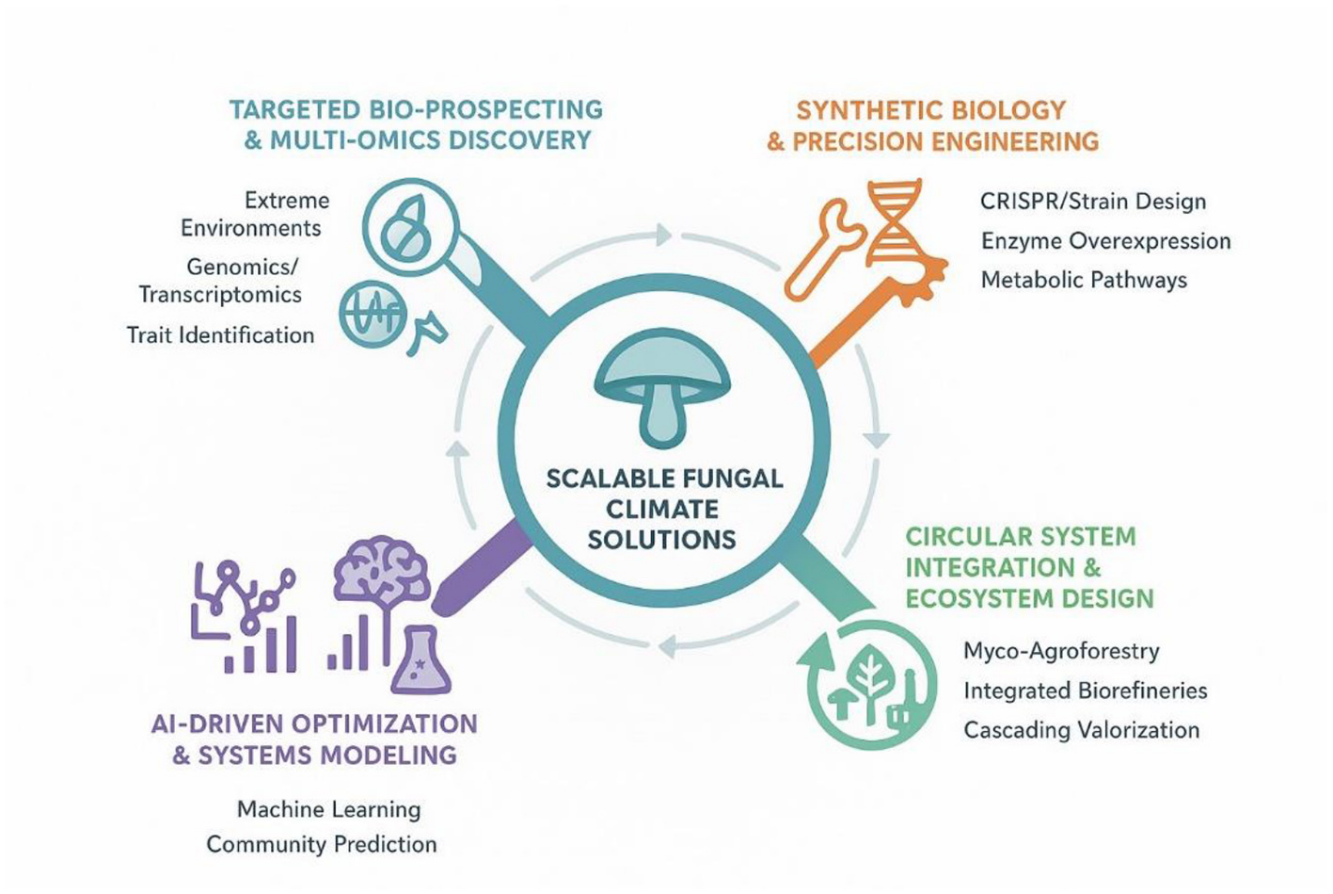
A conceptual diagram of the proposed convergent research framework, illustrating the four synergistic pathways, targeted bio-prospecting and multi-omics discovery, synthetic biology and precision engineering, ai-driven optimization and systems modeling, and circular system integration and ecosystem design, that interact to translate fungal potential into scalable climate solutions.

Our proposed framework (Figure 8) advocates for research that circulates between discovery, engineering, and systems integration via four key pathways. The first pathway is Targeted Bio-Prospecting and Multi-Omics Discovery. Future efforts must move beyond model species through systematic prospecting in understudied niches—such as marine and coastal ecosystems for blue-carbon associated Basidiomycetes, heavy metal-contaminated sites for hyper-tolerant strains, or ancient forests for novel mycorrhizal symbionts. Coupling this with genomics, transcriptomics, and proteomics will decode the genetic basis of desirable traits (e.g., high-affinity metal transporters, robust ligninolytic enzyme complexes, melanin biosynthesis pathways). This foundational knowledge is essential for identifying superior wild strains and providing genetic blueprints for the second pathway: Synthetic Biology and Precision Strain Engineering. To enhance and stabilize desired functions, the direct engineering of fungal metabolism is imperative. Key targets include the overexpression and secretion of lignin-modifying enzymes (laccases, peroxidases) in white-rot fungi for more efficient bioremediation and biomass pretreatment; the modulation of carbon allocation pathways in ectomycorrhizal fungi to favor the production of recalcitrant compounds like melanin and hydrophobins that enhance soil carbon persistence; and engineering substrate utilization networks in saprotrophs to efficiently convert heterogeneous waste streams into uniform mycelium for materials. Success in this pathway depends on parallel developments in fungal transformation tools, CRISPR-based editing, and addressing regulatory and public acceptance hurdles for engineered strains.

The third pathway, AI-Driven Optimization and Predictive Systems Modeling, addresses the complexity of fungal growth and environmental interactions, which are prime candidates for artificial intelligence. Machine learning algorithms can be trained to optimize substrate compositions and growth parameters for mycelium materials with bespoke mechanical properties; to predict the dynamics of fungal communities and their carbon cycling functions under different climate and land-use scenarios; and to design optimal fungal-bacterial consortia for synergistic pollutant degradation. Developing these models requires generating and curating large, high-quality, interdisciplinary datasets. Finally, the fourth pathway focuses on Circular System Integration and Ecosystem Design. The highest-impact applications will emerge from intentionally designing systems that leverage multiple fungal functions simultaneously. Research should prototype and test integrated models such as Myco-Agroforestry Systems, combining ECM-inoculated timber or nut trees for long-term carbon sequestration with understory cultivation of saprotrophic mushrooms on agricultural residues. Advanced Fungal Biorefineries, where lignocellulosic waste undergoes sequential valorization, first via fungal enzymatic pretreatment for bioethanol, followed by growth of edible mushrooms, and finally anaerobic digestion of the spent substrate, with the digestate returned to soil as a carbon-rich amendment.

### 6.3 Priority research questions emerging from the framework

This integrative logic leads to specific, high-priority research questions. First, can the co-cultivation of specific ECM fungi (e.g., *Pisolithus tinctorius*) and saprotrophic mushrooms (e.g., *Stropharia rugosoannulata*) in designed systems synergistically enhance total ecosystem carbon storage and soil health compared to single-function applications? Second, does targeted genetic enhancement of the melanin biosynthesis pathway in an ECM fungus (e.g., *Cenococcum geophilum*) measurably increase the mean residence time of fungal-derived carbon in mineral soils under field conditions? Third, what are the keystone metabolic interactions in a defined consortium of *Pleurotus ostreatus* and pollutant-degrading bacteria that maximize the mineralization of complex hydrocarbon mixtures, and can this consortium be stabilized in a low-cost, field-deployable bioreactor? Fourth, how do fungal community networks in soils respond to the large-scale application of spent mushroom substrate, and does this application ultimately lead to a net increase in stable mineral-associated organic carbon?

### 6.4 Overcoming policy and implementation hurdles

Technical advances must be coupled with enabling frameworks. Critical needs include developing standardized life-cycle assessment (LCA) methodologies to accurately quantify the carbon footprint and net sequestration benefits of fungal products and practices; creating policy incentives and carbon finance mechanisms that recognize and reward the ecosystem services provided by mycorrhizal networks and mycoremediation; and formally integrating mycorrhizal inoculation and fungal diversity metrics into reforestation guidelines, sustainable agriculture certification, and international climate accords like the Paris Agreement.

## 7 Conclusion

This review synthesizes extensive evidence that mushroom-forming fungi are far more than ecological components; they are versatile, living technologies integral to climate change mitigation. Their inherent capacities for long-term terrestrial carbon stabilization, powerful enzymatic remediation of pervasive pollutants, and production of biodegradable, carbon-neutral materials represent foundational pillars of the emerging sustainable bioeconomy. Yet, the path from recognizing this potential to realizing its impact at scale is neither straightforward nor assured.

The primary obstacle is a persistent translational gap between controlled laboratory studies and complex field applications. Our critical evaluation reveals that optimistic metrics for carbon sequestration or pollutant degradation are often derived from simplified systems that exclude key biotic interactions and environmental variabilities. Similarly, the promising properties of mycelium materials are frequently demonstrated under ideal conditions, not in the variable environments they are intended to replace. To bridge this gap, the field must evolve from a collection of proof-of-concept studies into a predictive, design-focused discipline. This necessitates a concerted shift toward field-relevant validation, experiments conducted in non-sterile soils, with contaminant mixtures, and under realistic climatic stresses—coupled with standardized life cycle and techno-economic assessments from the outset.

The true power of fungal solutions lies in their synergistic integration. The most transformative applications will not be single-technology fixes but designed, circular systems. Examples include myco-agroforestry systems that combine carbon-sequestering ectomycorrhizal trees with understory cultivation of saprotrophic mushrooms on agricultural residues, or advanced fungal biorefineries that cascade waste through sequential valorization for enzymes, food, bioenergy, and soil amendment. Developing these systems requires the interdisciplinary convergence outlined in our research framework, uniting targeted bioprospecting, synthetic biology, AI-driven optimization, and ecosystem design. To harness this potential within broader climate action, several actionable steps are imperative for researchers, industry, and policymakers. First, fungal processes must be formally integrated into climate policy and carbon accounting frameworks. The ecosystem services provided by mycorrhizal networks, such as enhanced soil carbon persistence and forest resilience, should be recognized and incentivized through carbon finance mechanisms and reforestation guidelines. Second, investment is urgently needed in pilot-scale facilities and long-term field trials to de-risk technologies, generate robust performance data, and create viable business models for mycoremediation and mycelium material production. Finally, fostering transdisciplinary collaboration is not optional; it is essential. Platforms must be created to unite mycologists with engineers, economists, and policymakers, ensuring fungal biotechnologies are developed with scalability, equity, and real-world impact as core principles. By embracing this integrative and applied roadmap, we can transition fungal biotechnology from a promising niche to a mainstream pillar of climate strategy. In doing so, we position these ancient and sophisticated organisms at the forefront of the essential transition from linear, extractive systems to resilient, regenerative, and truly circular economies, a necessary evolution for a stable and prosperous low-carbon future.
